# ERK1/2 inhibition reduces vascular calcification by activating miR-126-3p-DKK1/LRP6 pathway

**DOI:** 10.7150/thno.49771

**Published:** 2021-01-01

**Authors:** Peng Zeng, Jie Yang, Lipei Liu, Xiaoxiao Yang, Zhi Yao, Chuanrui Ma, Haibo Zhu, Jiamin Su, Qian Zhao, Ke Feng, Shu Yang, Yan Zhu, Xiaoju Li, Wenguang Wang, Yajun Duan, Jihong Han, Yuanli Chen

**Affiliations:** 1 College of Life Sciences, State Key Laboratory of Medicinal Chemical Biology, Key Laboratory of Bioactive Materials of Ministry of Education, Nankai University, Tianjin, China.; 2 Key Laboratory of Metabolism and Regulation for Major Diseases of Anhui Higher Education Institutes, Hefei University of Technology, Hefei, China.; 3 Tianjin Medical University, Tianjin, China.; 4 First Teaching Hospital of Tianjin University of Traditional Chinese Medicine, Tianjin, China.; 5 Chinese Academy of Medical Sciences and Peking Union Medical College, Beijing, China.; 6 Tianjin University of Traditional Chinese Medicine, Tianjin, China.; 7 Tianjin Chest Hospital, Tianjin, China.

**Keywords:** ERK1/2, miR-126-3p, vascular calcification, Wnt signaling, ECs

## Abstract

**Rationale**: Vascular microcalcification increases the risk of rupture of vulnerable atherosclerotic lesions. Inhibition of ERK1/2 reduces atherosclerosis in animal models while its role in vascular calcification and the underlying mechanisms remains incompletely understood.

**Methods:** Levels of activated ERK1/2, DKK1, LRP6 and BMP2 in human calcific aortic valves were determined. ApoE deficient mice received ERK1/2 inhibitor (U0126) treatment, followed by determination of atherosclerosis, calcification and miR-126-3p production. C57BL/6J mice were used to determine the effect of U0126 on Vitamin D_3_ (VD_3_)-induced medial arterial calcification. HUVECs, HAECs and HASMCs were used to determine the effects of ERK1/2 inhibitor or siRNA on SMC calcification and the involved mechanisms.

**Results**: We observed the calcification in human aortic valves was positively correlated to ERK1/2 activity. At cellular and animal levels, U0126 reduced intimal calcification in atherosclerotic lesions of high-fat diet-fed apoE deficient mice, medial arterial calcification in VD_3_-treated C57BL/6J mice, and calcification in cultured SMCs and arterial rings. The reduction of calcification was attributed to ERK1/2 inhibition-reduced expression of ALP, BMP2 and RUNX2 by activating DKK1 and LRP6 expression, and consequently inactivating both canonical and non-canonical Wnt signaling pathways in SMCs. Furthermore, we determined ERK1/2 inhibition activated miR-126-3p production by facilitating its maturation through activation of AMPKα-mediated p53 phosphorylation, and the activated miR-126-3p from ECs and SMCs played a key role in anti-vascular calcification actions of ERK1/2 inhibition.

**Conclusions**: Our study demonstrates that activation of miR-126-3p production in ECs/SMCs and interactions between ECs and SMCs play an important role in reduction of vascular calcification by ERK1/2 inhibition.

## Introduction

Vascular calcification affects aortic rigidity which results in hypertension, left ventricular hypertrophy, ischemia, heart failure and death 1. Nearly all patients with cardiovascular disease have some degree of calcification 2. Calcific vasculopathy occurs to the intima (within atherosclerotic plaque) and/or the tunica media [commonly associated with chronic kidney disease (CKD)]. Intimal microcalcification, such as the spotty-shaped calcification in atherosclerotic lesions, can reduce lesion stability thereby increasing the risk of plaque rupture 3, 4. Clinically, the mortality in patients with high coronary artery calcium scores is substantially increased 5.

Smooth muscle cells (SMCs) are a pluripotent cell type in vasculatures and present as the contractile phenotype physiologically. SMCs are the main source of vascular osteoblast differentiation which contributes to vascular calcification and status of different kinds of diseases, such as atherosclerosis, hypertension, cardiovascular complications in diabetic mellitus/CKD and myocardial infarction 2, 6. The signaling molecules of Wnt, transforming growth factor β, bone morphogenetic proteins (BMPs) and Notch are important regulators of ectopic calcification 7. For instance, absence of Wnt inhibitors, such as dickkopf 1 (DKK1), results in β-catenin nuclear translocation and consequent induction of osteogenic gene expression 8, which indicates the involvement of DKK1 in ectopic calcification. Indeed, circulating DKK1 is negatively associated with aortic calcification scores 9-11. Moreover, the interaction between endothelial cells (ECs) and SMCs also contributes to SMC osteogenesis. EC-derived inflammatory cytokines, chemokines, adherent molecules and other types of molecules can regulate SMC phenotypic switch and calcification 12-14, thereby profoundly influencing vascular structural integrity.

microRNAs (miRNAs), a large family of small noncoding RNAs, regulate target gene expression at the post-transcriptional level. They play an important role in various diseases including cardiovascular disease. miR-126-3p is located in the intron of EGF like domain multiple 7 (EGFL7) gene and predominantly produced by ECs 15, 16. Maturation of miR-126 generates two functional miRNAs, namely miR-126-3p (miR-126) and miR-126-5p (miR-126*). miR-126-3p has been demonstrated anti-atherogenic properties by regulating different pathways 17, 18. For instance, miR-126-3p delivered by EC apoptosis bodies inhibits expression of regulator of G-protein signaling 16 (RGS16) to activate C-X-C motif chemokine ligand 12 (CXCL12) and its receptor, C-X-C motif chemokine receptor 4 (CXCR4), thereby reducing atherosclerosis 18. miR-126-3p also inhibits adhesion of leukocytes to ECs by reducing vascular cell adhesion molecule-1 (VCAM-1) 19.

Extracellular signal-regulated kinases 1/2 (ERK1/2), also known as p42/44 mitogen-activated protein kinase (p42/44 MAPK), are enzymes involved in various biological processes 20. ERK1/2 are the specific substrates for MAPK kinase 1 and 2 (MEK1/2). Thus, inhibitors of MEK1/2, such as PD98059 and U0126, are also referred to as ERK1/2 inhibitors. Regulation of ERK1/2 activity (the phosphorylated ERK1/2 or p-ERK1/2 is the active form of ERK1/2) is implicated in cardiovascular disease 21-25. Reduced ERK1/2 activity by inhibitors increases elastin synthesis and aortic elastin content, indicating the feasibility of ERK1/2 inhibitors in treating vascular pathology with diminished arterial elastin content 22. Previous studies have demonstrated the involvement of ERK1/2 in vascular calcification. For instance, mice with a germline deletion of ERK1 and a conditional deletion of ERK2 in limb mesenchyme (ERK1^-/-^ERK2^Prx1^ mice), including osteoblasts, display substantially reduced bone mineralization, suggesting the importance of ERK for osteoblast mineralization 21. Further studies indicate ERK1/2 mediates RUNX2 phosphorylation directly at four sites, S43, S301, S319, and S510, among which S301 and S319 each contributes to RUNX2 transcriptional activity 26, 27. Moreover, ERK2 directly binds and phosphorylates CK2α at T360/S362, which subsequently enhances CK2α activity toward α-catenin phosphorylation. Phosphorylation of α-catenin results in disruption of the complex of β-catenin and α-catenin, thereby abrogating the inhibitory effect of α-catenin on β-catenin transactivation 28. We previously reported that ERK1/2 inhibition synergizes liver X receptor (LXR)-reduced atherosclerosis in apoE deficient (apoE^-/-^) mice by enhancing reverse cholesterol transport and ATP-binding cassette transporter A1 (ABCA1) expression 23. ERK1/2 inhibition also activates macrophage ABCG1 expression to inhibit macrophage/foam cell formation 29. Our findings of anti-atherogenic properties of ERK1/2 inhibition have been confirmed by a recent report, which demonstrates protection against hypercholesterolemia and atherosclerosis by deficiency of orphan G protein-coupled receptor 146 (GPR146) expression is tightly linked to reduced ERK1/2 activity 30. In spite of above findings, the detailed anti-atherogenic actions and the underlying mechanisms by ERK1/2 inhibition, particularly the effect and detailed mechanisms on vascular calcification and the interactions between ECs and SMCs, need more investigation.

## Materials and methods

The data that support the findings of this study are available from the corresponding authors on reasonable request.

The detailed method section is available in the **Supplementary materials**.

### Cell culture

EA.hy926 cells (purchased from ATCC), a human umbilical vein cell line, were cultured in DMEM medium containing 10% fetal bovine serum (FBS), 50 µg/mL penicillin/streptomycin. Human umbilical vein endothelial cells (HUVECs) and human aorta endothelial cell (HAEC) were purchased from ATCC and cultured in Vasculife basal medium (EC medium) containing VEGF Lifefactors kit (complete EC medium, Lifeline Cell Technology, Frederick, MD, USA). Human aortic smooth muscle cells (HASMCs) were kindly provided by Dr. Xi-long Zheng from Calgary University in Canada and cultured in DMEM/F12 (V : V = 1 : 1) medium supplemented with 10% FBS, 50 µg/mL penicillin/streptomycin and 2 mM glutamine. HASMCs less than 8 passages were used for experiments. ECs received indicated treatment in serum-free medium.

### Determination of calcification in human calcific aortic valve samples

The study with human samples was approved by the Ethical Review Board of Tianjin Chest Hospital (Tianjin, China) and adhered strictly to the Declaration of Helsinki Principle 2008. All samples were collected after the informed written consents were signed by patients and their family members.

Transthoracic echocardiography diagnostic criteria for aortic valve calcification was leaf thickness > 2 mm and local echo enhancement in aortic valve 31. Dual source computed tomography (DSCT) diagnostic criteria was that lesions were founded in aortic valves or in aortic root and the density was more than 130 HUs in 3 or more consecutive pixel 32. The double positive patients (~15) were finally recruited *per* inclusion and exclusion criteria, and they were assigned as calcific aortic valve disease (CAVD).

The resected valve sample was further divided into two parts: calcification area (white and firm tissues, confirmed by von Kossa staining) and adjacent area (brownness and soft tissues), followed by determination of BMP2, RUNX2, DKK1 and LRP6 mRNA expression by qRT-PCR with total RNA extract. The calcification and expression of p-ERK1/2 and RUNX2 protein were determined by Alizarin Red S and immunofluorescent staining 23, with quantitation of the correlation coefficient between RUNX2 MFI and p-ERK1/2 MFI.

### *In vivo* studies with mice

The protocols for *in vivo* study with mice were approved by the Ethics Committee of Nankai University and conform to the Guide for the Care and Use of Laboratory Animals published by NIH.

ApoE deficient (apoE^-/-^) and C57BL/6J mice were obtained from Gempharmatech Co., Ltd. (Nanjing, Jiangsu, China). All mice were housed in the Animal Center of Nankai University (Tianjin, China). To study the effect of U0126 on atherosclerosis and vascular calcification in lesion areas, apoE^-/-^ mice were randomly divided into two groups (15 mice/group), and fed a proatherogenic high-fat diet (HFD, 21% fat and 0.5% cholesterol) or HFD containing U0126 (U0, 3 mg/kg bodyweight/day), respectively. After 16 weeks of treatment, mouse aorta and serum samples were collected individually 23.

In order to determine the effect of U0126 on Vitamin D_3_ (VD_3_)-induced medial arterial calcification, C57BL/6J mice (male, 16-week, n = 6) were randomly divided into control group (sham), medial arterial calcification model group (model), and medial arterial calcification model receiving U0126 treatment group (U0126). Medial arterial calcification model was induced by s.c injection of 100 µL VD_3_ (5.5×10^5^ U/kg) once a day for three times as described 33, 34. The mice subjected to model induction were then fed chow diet or chow diet containing U0126 (3 mg/kg bodyweight/day) for 6 weeks. At the end of the experiment, mice were anesthetized and euthanized to collect blood and aorta samples.

### Determination of lesions and calcification

The entire aorta and aortic root cross sections were prepared and used to determine lesions as described 23. Atherosclerotic lesions in *en face* aorta or cross sections of aortic root were calculated according to the guidelines for experimental atherosclerosis studies described in the American Heart Association Statement 35. The *en face* or sinus lesions were quantified by 2 persons who were blinded to the experimental design and each other's results using a computer-assisted image analysis protocol. Lesions in *en face* aorta or cross sections of aortic root are expressed as mean percentage of lesion areas in the aorta or lumen area ± SD.

Vascular calcification formed* in vivo* or *ex vivo* was determined by Alizarin Red S and von Kossa staining of aortic root or thoracic aorta cross sections, whole-mount artery, or aortic rings, and calcium quantitative assay. *In vitro*, HASMCs were induced calcification by culture in complete DMEM/F12 medium (1 : 1) containing 10 mM β-glycerol phosphate and 250 µM ascorbic acid or plus treatment for 7 days, followed by Alizarin Red S staining and calcium quantitative assay 36.

### Determination of protein expression by immunofluorescent staining and Western blot

Expression of BMP2, RUNX2, DKK1, LRP6, β-catenin, OPN, VCAM-1, p-ERK1/2, ERK1/2 and Ki-67 in aortic root, BMP2, RUNX2, p-ERK1/2 and ERK1/2 in thoracic artery, and β-catenin, RUNX2, BMP2 and ALP in HASMCs were determined by immunofluorescent staining of the 5-µm frozen sections or the slides of cell culture and primary antibodies. The negative control immunofluorescent staining was conducted using normal IgG to replace primary antibody. After the images were captured, the mean fluorescent intensity (MFI) of immunofluorescent image was calculated as described 37. Expression of target proteins were determined by Western blot with total or nuclear proteins by Western blot as described 38.

### Transfection of siRNA and determination of mRNA and miRNA

HUVECs, HAECs and HASMCs in 6-well plates (for Western blot or qRT-PCR) or 24-well plates (for Alizarin Red S staining) were transfected with siRNA, miR-126-3p mimic or antagomir using Lipofectamine^®^ RNAiMAX Transfection Reagent (Invitrogen). The efficiency for all siRNA was validated by Western blot and qRT-PCR, and the concentrations of siRNA used in this study had no effect on cell viability as determined by MTT assay (Figure S15).

Expression of EGFL7, ERK1, ERK2, AMPKα1, AMPKα2, and p53 mRNA in HUVECs, and ERK1, ERK2, USF1, RUNX2, Osx, SOX9, DKK1, LRP5 and LRP6 mRNA in HASMCs were determined by quantitative real time RT-PCR (qRT-PCR) with total RNA extracted from tissue or cell samples and primers with sequences listed in Table S1. Levels of miR-126-3p in serum, tissue, cells or EC-conditioned medium and miR-145, miR-143 and miR-155 in cells were analyzed by quantitative miR stem-loop RT-PCR technology (Ambion) 18.

### Collection and treatment of the EC-conditioned medium

After treatment, HUVECs or HAECs in 100-mm dishes were washed twice with PBS, and continued culture in serum-free EC medium for 24 h. The 24-h EC-conditioned medium was collected individually. To inactivate secreting miRNA complex, the collected EC-conditioned medium was treated with proteinase K, RNase or both, followed by determination of miR-126-3p levels and used to determine the effect on HASMC calcification.

### Data analysis

Data were generated from at least three independent experiments. Values are represented as mean ± SD. The raw data were initially subject to a normal distribution analysis with SPSS software (1-sample K-S of non-parametric test). All the data in normal distribution were then analyzed by the parametric statistics (Graph Pad Prism 7.0 software), unpaired Student's T-test (two groups) or one-way ANOVA followed by Tukey's* post hoc* test (more than two groups). The correlation between human valve RUNX2 and p-ERK1/2 was estimated by Pearson's method. *P* < 0.05 was considered significant.

## Results

### Activated ERK1/2 is positively correlated to calcification in human calcific aortic valves

**Figure 1:**
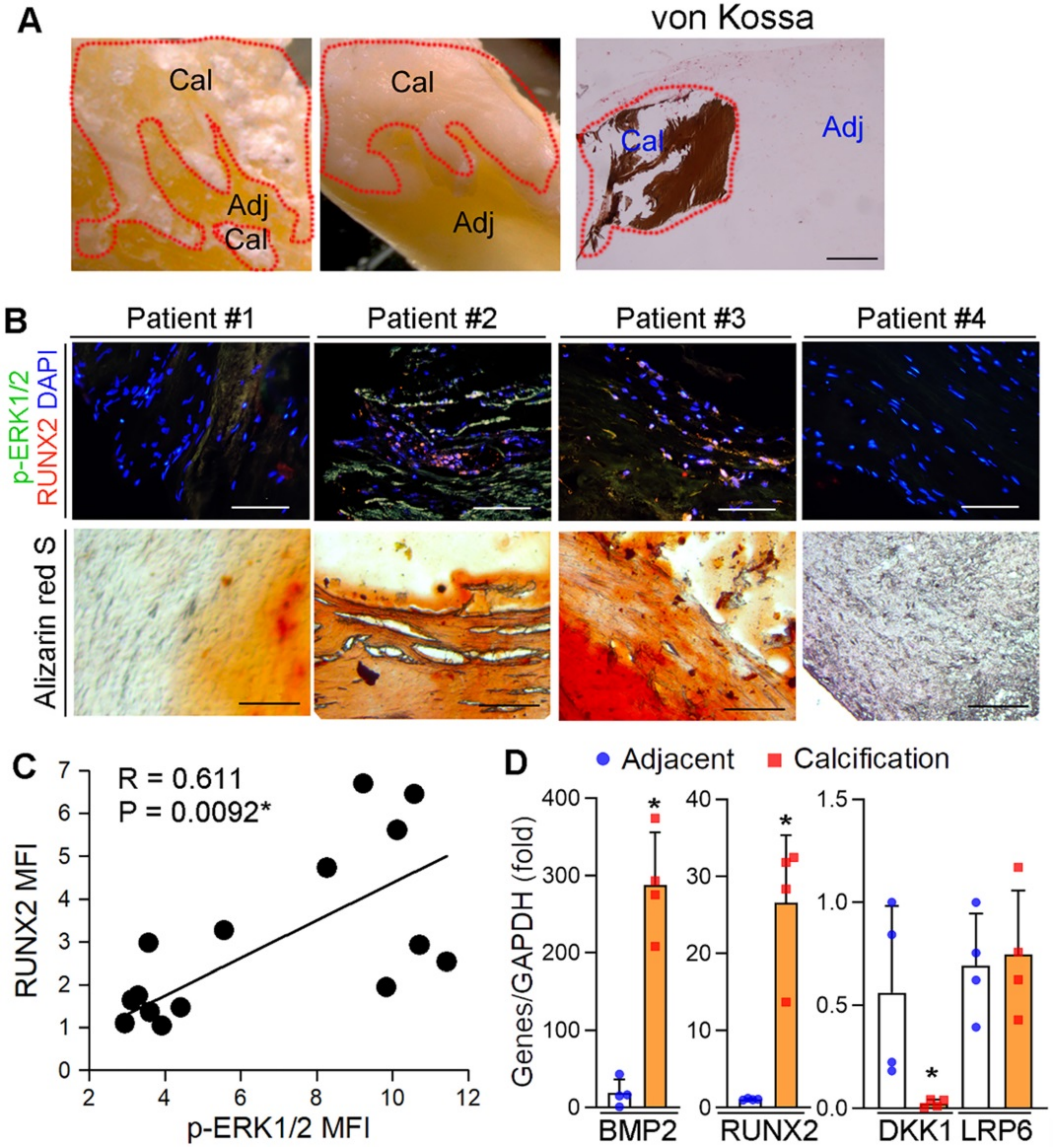
** Activated ERK1/2 is positively correlated to calcification in human calcific aortic valves.** (**A**) The resected aortic valve samples were collected from CAVD patients (n = 15). The sample was divided into two parts: calcification and adjacent areas. The calcification was initially confirmed by von Kossa staining. Bar: 200 µm. (**B-C**) the paraffin sections of aortic valves were prepared and used to determine calcification by Alizarin Red S staining and expression of p-ERK1/2 and RUNX2 by immunofluorescent staining. The images from 4 representative samples were presented. Bars: 200 µm. (**D**) the calcification and adjacent areas were collected from samples in Figure 1A and extracted total RNA, followed by determination of BMP2, DKK1, LRP6 and RUNX2 mRNA expression by qRT-PCR. *P < 0.05 (n = 4).

We previously reported the protection of ERK1/2 inhibitors against atherosclerosis by improving macrophage cholesterol metabolism through induction of ABCA1/G1 and interleukin-5 expression 23, 29, 39. However, the mechanisms of ERK1/2 inhibitor-increased lesion stability, especially the effect and mechanisms on ectopic calcification, was incompletely understood. To determine the role of ERK1/2 activity in ectopic calcification, calcific aortic valve samples were collected by surgical resection from 15 patients with calcific aortic valve disease (CAVD) (the basal clinical characteristics of the patients was presented in Table S2). Calcification was initially confirmed by von Kossa staining (Figure 1A). The sample sections were determined calcification and expression of activated ERK1/2 (p-ERK1/2) and Runt related transcription factor 2 (RUNX2). p-ERK1/2 was high in Alizarin Red S positive areas (Figure 1B), suggesting activated ERK1/2 is positively correlated to calcification. Figure 1B-C also demonstrate the activated ERK1/2 is positively correlated to RUNX2 expression (R = 0.611, *P =* 0.0092). In addition, expression of pro-calcification genes, bone morphogenetic protein 2 (BMP2) and RUNX2, were increased, while expression of dickkopf 1 (DKK1), an anti-calcification molecule, was reduced in human calcified aortic valves (Figure 1D).

### Inhibition of ERK1/2 reduces vascular calcification

**Figure 2:**
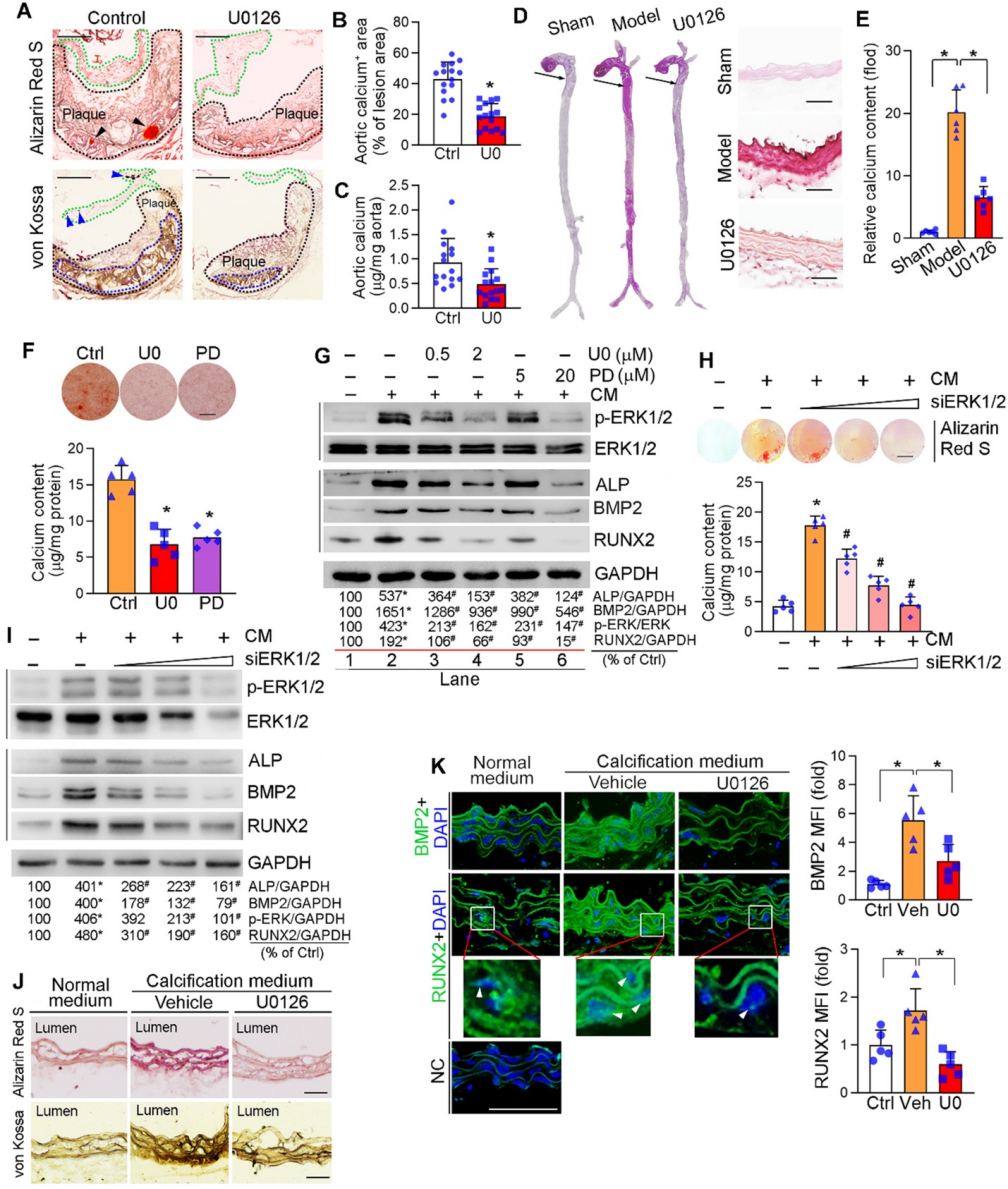
** Inhibition of ERK1/2 by inhibitor/siRNA reduces calcification.** (**A**) apoE^-/-^ mice (15 mice/group) were fed HFD or HFD containing U0126 (3 mg/day/kg bodyweight) for 16 weeks. Aortic root cross sections were used to determine calcification formed within aortic root lesion area by Alizarin Red S (top panel) and von Kossa staining (bottom panel) (black dash lines: lesion area; green dash lines: aortic valve; black arrowheads and blue dash lines: calcification). Bars: 200 µm. (**B**) the percentage of calcium positive area in whole aortic lesion area was quantitatively analyzed based on images in the bottom panel of Figure 2A. *P < 0.05 (n = 15). (**C**) Total calcium of aorta was extracted and determined by an assay kit. *P < 0.05 (n = 15). (**D-E**) C57BL/6J mice (6 mice/group) were s.c. injected with 100 µL olive oil (Sham) or VD_3_ (5.5x10^5^ U/kg) dissolved in olive oil every day for three days, followed by feeding chow diet (Model) or chow diet containing U0126 (3 mg/kg bodyweight/day) (U0126) for 6 weeks. The whole artery and thoracic aorta (indicated by black arrows) cross sections were used to determine calcification by Alizarin Red S staining (**D**) and calcium quantitatively analysis (**E**), bars: 50 µm. (**F-G**) HASMCs were cultured in complete DMEM/F12 (1 : 1) medium or calcification medium (CM). Cells in CM were also treated with U0126 (U0) or PD98059 (PD) at the indicated concentrations (F: 2 µM U0126; 20 µM PD98059). After 7 days of calcification induction or plus treatment, cells were conducted Alizarin Red S staining and calcium quantitative assay (n = 5). Bars: 10 mm (F). Total cellular proteins were extracted and determined expression of p-ERK1/2, ERK1/2, ALP, BMP2 and RUNX2 by Western blot, *P < 0.05 *vs.* lane 1; ^#^P < 0.05 *vs.* lane 2, n = 3 (G). (**H-I**) HASMCs were transfected with ERK1/2 siRNA (siERK1/2: 5, 20, 40 nM) for 24 h, followed by culture in complete DMEM/F12 (1 : 1) medium or CM for 7 days. Cells were then conducted Alizarin Red S staining and calcium quantitative assay (H, n = 5), Bars: 5 mm. Total cellular proteins were used to determine expression of p-ERK1/2, ERK1/2, ALP, BMP2 and RUNX2 by Western blot, *P < 0.05 *vs.* lane 1; ^#^*P* < 0.05 *vs.* lane 2, n = 3 (I). (**J-K**) thoracic aorta was collected from apoE^-/-^ mouse and cut into 5-mm long aortic rings. After cultured in complete DMEM/F12 medium, CM or CM plus U0126 (2 µM) for 14 days, rings were used to prepare 5-µm frozen sections. Calcium deposit and expression of BMP2 and RUNX2 in aortic rings were determined by Alizarin Red S and von Kossa staining (J), immunofluorescent staining (K) with quantitation of BMP2 and RUNX2 MFI, respectively. White arrow head indicated the nuclear RUNX2. Bars: 50 µm.

Intimal microcalcification is a risk factor for rupture of vulnerable lesion plaques due to reduced lesion stability 3-5. Owing to some common pathological mechanisms between aortic valve calcification and vascular calcification, such as activation of Wnt signaling pathway, alkaline phosphatase (ALP), BMP2 and RUNX2 40-42, the association between activated ERK1/2 and aortic valve calcification in human samples implies ERK1/2 inhibitor, such as U0126, can reduce vascular calcification. To determine it, high-fat diet (HFD)-fed apoE^-/-^ mice received U0126 oral treatment (mixed with HFD) for 16 weeks. Similar to our previous study, U0126 alone reduced atherosclerotic lesions, but it had no effect on mouse body weight gain and serum lipid profiles (Figure S1A-D), indicating the anti-atherosclerosis by ERK1/2 inhibitor is un-related to amelioration of hypercholesterolemia. The long-term U0126 treatment significantly inhibited p-ERK1/2 without effect on total ERK1/2 levels in lesion areas, indicating U0126 can attenuate HFD-activated ERK1/2 *in vivo* (Figure S1E). Meanwhile, Alizarin Red S and von Kossa staining showed that U0126 reduced calcium accumulation in aortic valve and atherosclerotic lesions of apoE^-/-^ mouse aortic root cross sections (Figure 2A-B), suggesting its anti-calcification functions. The reduction of vascular calcification by U0126 was further confirmed by quantitative analysis of calcium content in aortas (Figure 2C).

We also used the VD_3_-induced medial arterial calcification model with C57BL/6J mice to further disclose the inhibitory effect of ERK1/2 inhibition on vascular calcification. Subcutaneous (s.c.) injection of VD_3_ or oral administration of U0126 had little effect on mouse body weight gain (Figure S2A). However, VD_3_ significantly increased serum urea nitrogen and alanine aminotransferase (ALT) levels, indicating it causes some metabolic disorders in tissues. ERK1/2 inhibition restored both urea nitrogen and ALT to normal (Figure S2B-C). However, ERK1/2 inhibition had little effect on serum lipid profiles except reducing TG level moderately (Figure S2D). The VD_3_-increased p-ERK1/2 was abolished by U0126 (Figure S2E). Interestingly, U0126 also reduced calcium accumulation in VD_3_-treated mouse aorta and thoracic aorta cross sections (Figure 2D), further demonstrating the anti-calcification functions of ERK1/2 inhibitor. Quantitation of calcium content in aortas also confirmed the reduction of vascular calcification by U0126 in this medial arterial calcification model (Figure 2E).

Vascular calcification mainly occurs to vascular SMCs. We initially observed that treatment of human aortic SMCs (HASMCs) with ERK1/2 inhibitor or siRNA reduced cellular calcium deposit induced by a calcification medium (the complete DMEM/F12 medium containing 10 mM β-glycerol phosphate and 250 µM ascorbic acid, and named as “CM”) determined by Alizarin Red S staining and calcium quantitative assay (Figure 2F, H). ERK1/2 inhibitors also reduced HASMC calcification induced by inorganic phosphate [DMEM/F12 medium containing 3 mM Na_2_HPO_4_/NaH_2_PO_4_ (1 : 2)] (Figure S3).

Calcification in SMCs also activated p-ERK1/2 without affecting total ERK1/2 expression. Both ERK1/2 inhibitor and siRNA reduced activated ERK1/2 (p-ERK1/2) in a concentration-dependent manner (top panels in Figure 2G, I). Associated with increased p-ERK1/2, CM induced expression of vascular calcification markers, such as ALP and BMP2. While decreased p-ERK1/2 by inhibitor or siRNA reduced ALP and BMP2 expression in HASMCs in a concentration-dependent manner (middle panels in Figure 2G, I). RUNX2 is the transcription factor activating osteoblast differentiation and ALP/BMP2 expression 43. Both RUNX2 protein and transcript were activated by CM, but the activation was blocked by ERK1/2 inhibition (middle panels of Figure 2G, I; Figure S4A). Similarly, expression of another transcription factor for osteoblast differentiation, osterix (Osx), was activated by CM but blocked by U0126 (Figure S4B). In contrast, U0126 had no effect on calcification-induced expression of sex-determining region Y-box 9 (SOX9), the transcription factor controlling chondrocyte differentiation (Figure S4C). Thus, the data above also demonstrate the positive correlation between ERK1/2 activity and calcification in SMCs *in vitro*.

The anti-calcification function of ERK1/2 inhibition was further confirmed by an *ex vivo* study. The thoracic aorta was collected from apoE^-/-^ mice and cut into 5-mm long rings, followed by induction of vascular calcification. Figure 2J shows CM clearly induced calcium deposit in aortic rings, but the induction was blocked by U0126. Accordingly, expression of BMP2 and RUNX2 were dramatically increased by CM, and the increases were blunted by U0126 (Figure 2K). In addition, ERK1/2 inhibition reduced expression of BMP2 and RUNX2 (total and nucleic) in arterial wall of the two calcification mouse models (Figure S4D-E). Taken together, Figure 2, S3 and S4 suggest that ERK1/2 inhibition reduces vascular calcification and expression of osteogenic genes.

Furthermore, we excluded the off-target and cytotoxic effects of ERK1/2 inhibition in anti-vascular calcification. Compared to reduced p-ERK1/2 (Figure 2G, I), neither phosphorylated p38 MAPK (p-p38 MAPK) nor phosphorylated JNK1/2 (p-JNK1/2) in SMCs was affected by ERK1/2 inhibitor or siRNA (Figure S5). We also determined that both U0126 and PD98059 had little effect on viability, cycle and apoptosis of HASMCs or human umbilical vein endothelial cells (HUVECs), except the G1/S transition in HUVECs was moderately inhibited (Figure S6). *In vivo*, U0126 increased SMC proliferation while did not affect EC cell proliferation or SMC and EC apoptosis in mouse aortic plaque (Figure S7). Taken together, the results above suggest that ERK1/2 inhibitors have little effect on cell apoptosis while moderately enhances SMC proliferation *in vivo*, indicating a high safety used at a low dose/concentration range *in vivo* and *in vitro*.

### ERK1/2 inhibition reduces vascular calcification by inactivating canonical and non-canonical Wnt signaling pathway

**Figure 3:**
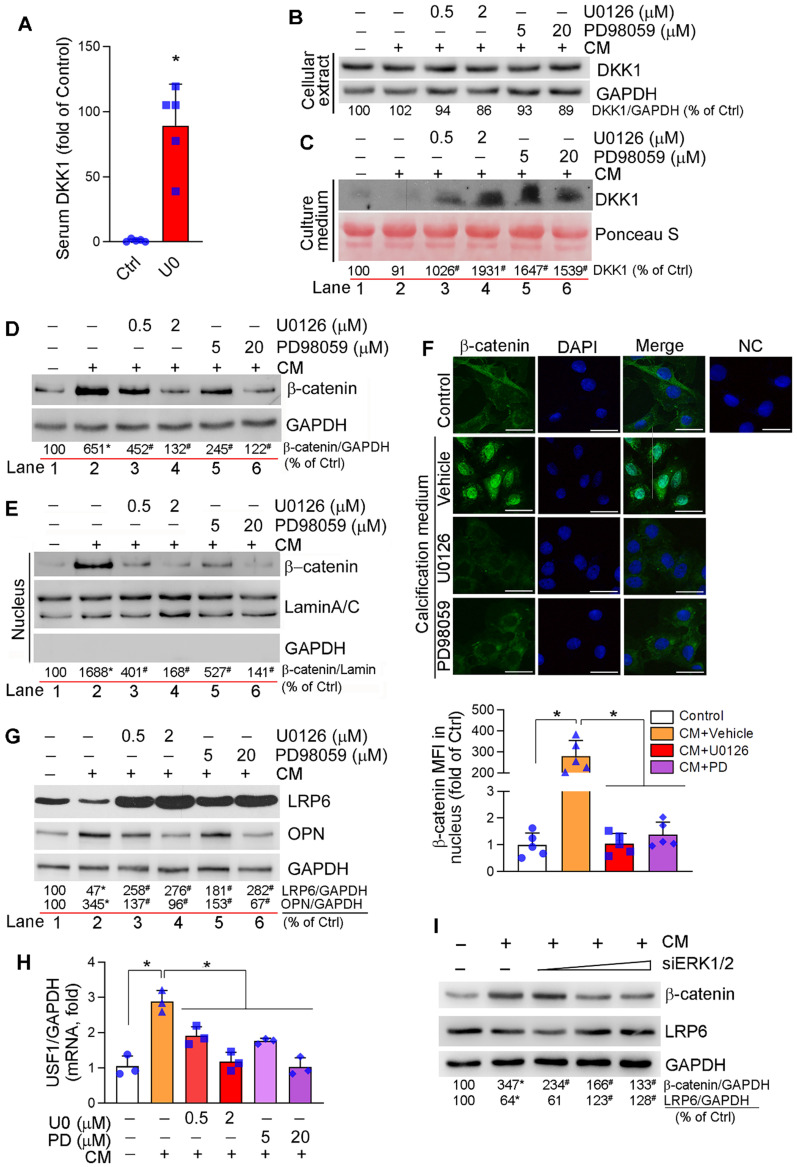
** Regulation of canonical and non-canonical Wnt signaling pathways by ERK1/2 inhibition.** (**A**) DKK1 levels in serum samples collected from mice in Figure 2A were determined by the “Antibody Arrays” method. The mean DKK1 level in control mice was defined as 1. *P < 0.05 (n = 5). (**B-H**) HASMCs were cultured in complete DMEM/F12 medium, CM or CM plus U0126/PD98059 at the indicated concentrations for 7 days, and then completed the following assays: expression of DKK1 (**B**), β-catenin (**D**), LRP6 and OPN (G) in total cellular proteins, DKK1 in concentrated HASMC-conditioned medium (**C**) and nuclear β-catenin (**E**) were determined by Western blot. *P < 0.05 *vs.* lane 1; ^#^*P* < 0.05 *vs.* lane 2 (n = 3). Ponceau S staining was conducted as the loading control of the HASMC-conditioned medium. Expression of β-catenin in HASMCs was determined by immunofluorescent staining (**F**) with quantitation of β-catenin MFI in nucleus. Bars: 20 µm. Expression of USF1 mRNA was determined by qRT-PCR (**H**). *P < 0.05 (n = 3). (**I**) HASMCs were transfected with ERK1/2 siRNA (siERK1/2: 5, 20, 40 nM) for 24 h, followed by culture in complete DMEM/F12 (1 : 1) medium or CM for 7 days. Total cellular proteins were used to determine expression of β-catenin and LRP6 by Western blot, *P < 0.05 *vs.* Control; ^#^*P* < 0.05 *vs.* CM control (n = 3).

DKK1, a secreting protein, inhibits canonical Wnt or Wnt/β-catenin signaling pathway by competitively binding and inhibiting Wnt receptors, LRP5 and LRP6 44. Clinically, serum DKK1 levels are negatively correlated to arterial calcification and atherosclerotic plaque formation 9-11. We found the long-term U0126 treatment increased serum DKK1 levels (Figure 3A) and DKK1 expression in aortic lesion area of HFD-fed apoE^-/-^ mice (Figure S8A). Calcification had little effect on DKK1 mRNA (Figure S8B) and protein expression/secretion in HASMCs (Figure 3B-C). However, ERK1/2 inhibitors activated DKK1 mRNA expression (Figure S8B) and DKK1 protein secretion (Figure 3C) which is consistent to increased serum DKK1 levels *in vivo* (Figure 3A). However, ERK1/2 inhibitor had no effect on cellular DKK1 protein levels (Figure 3B), given DKK1 is a secreting protein.

Either canonical or non-canonical Wnt signaling pathway is involved in osteoblast differentiation 45, 46. Calcification activated HASMC β-catenin expression, which was reduced by ERK1/2 inhibitor or siRNA in a concentration-dependent manner (Figure 3D, I). Furthermore, increased β-catenin expression was determined on day 4 of calcification induction with a peak of expression between day 7-14, and the induction was inhibited by U0126 treatment throughout (Figure S8E). *In vivo*, inhibition of β-catenin expression by U0126 was observed in aortic root cross sections (Figure S8A).

Activation of Wnt/β-catenin signaling pathway induces β-catenin nuclear translocation, where it binds to TCF/LEF transcription factors and activates expression of osteogenic genes including RUNX2, osterix and ALP 45. Calcification induced both β-catenin expression and nuclear translocation in HASMCs, but the induction was substantially blocked by ERK1/2 inhibition (Figure 3D-F). These data suggest ERK1/2 inhibition inactivates canonical Wnt signaling pathway.

Although the association of LRP6 with Wnt ligands activates canonical Wnt signaling pathway by mediating β-catenin nuclear translocation, LRP6 has been demonstrated its potent inhibitory effect on non-canonical Wnt signaling pathway to suppress expression of upstream stimulatory factor 1 (USF1) and osteopontin (OPN) 46. Therefore, deficiency of SMC LRP6 expression induces severe calcification in atherosclerotic lesions 46. LRP6 expression in HASMCs was reduced by calcification. However, LRP6 was increased dramatically by both ERK1/2 inhibitor and siRNA in a dose-dependent manner in HASMCs (Figure 3G, I; S8C). Associated with changes of LRP6 expression regulated by calcification or ERK1/2 inhibition, expression of OPN protein and USF1 mRNA was inversely regulated (Figure 3G-H). In contrast, U0126 slightly reduced calcification-induced LRP5 mRNA expression (Figure S8D). *In vivo*, induction of LRP6 and inhibition of OPN expression by U0126 were observed in aortic root cross sections (Figure S8A). The results above suggest that reduction of vascular calcification by ERK1/2 inhibition is also linked to inactivation of non-canonical Wnt signaling pathway by activating LRP6.

**Figure 4:**
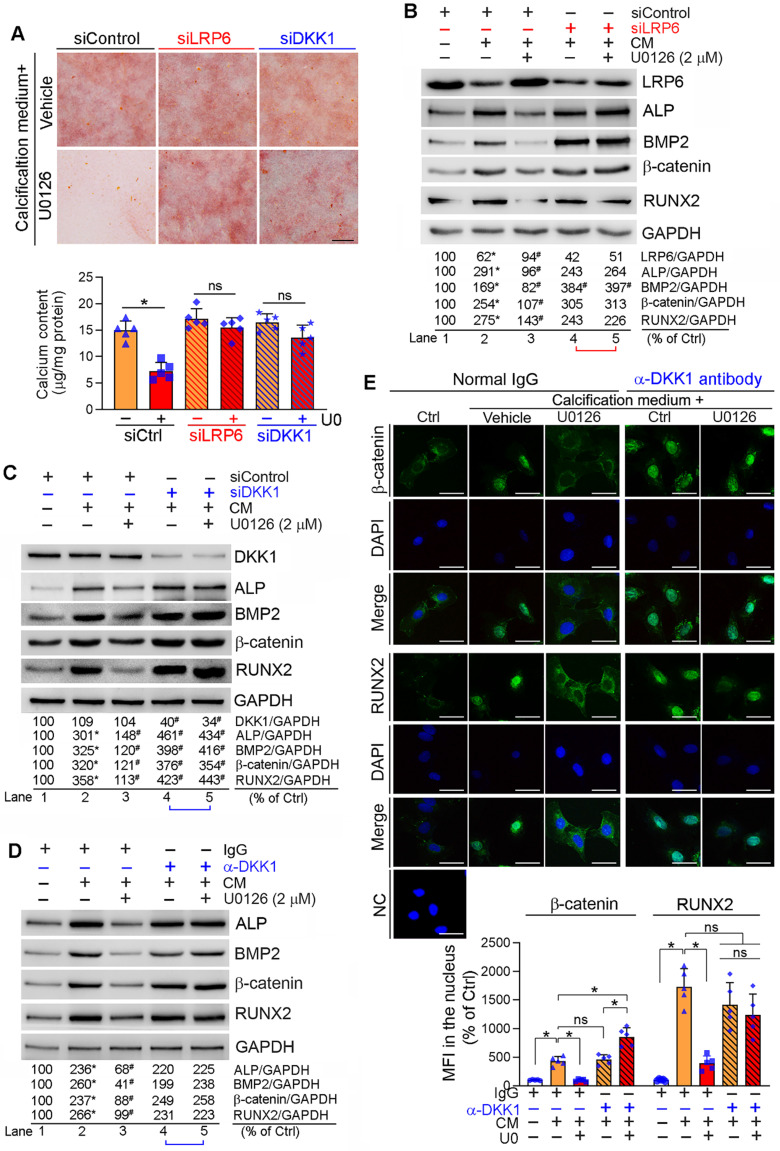
** Inhibition of DKK1 or LRP6 expression abolishes ERK1/2 inhibition-inhibited calcification in HASMCs.** HASMCs were induced calcification by culture in CM or CM plus the following treatment for 7 days: (**A-C**) HASMCs were transfected with siRNA against human LRP6 (siLRP6) or DKK1 (siDKK1) for 24 h. Cells were then switched to complete DMEM/F12 medium, CM or CM plus U0126 (2 µM). The siRNA transfection was repeated once on day 3 of the calcification induction. Bars: 0.5 mm; (**D-E**) HASMCs were cultured in complete DMEM/F12 medium, CM or CM containing normal IgG or anti-DKK1 antibody or plus U0126 (2 µM) as indicated for 7 days. At the end of experiments, cells were used for the following assays: (**A**) calcium deposit by Alizarin Red S staining and calcium quantitative assay. *P < 0.05 *vs*. CM alone, n = 3; (**B-D**) expression of BMP2, β-catenin, RUNX2, ALP, DKK1 and LRP6 by Western blot. *P < 0.05 *vs.* lane 1; ^#^*P* < 0.05 *vs.* lane 2 (n = 3). (**E**) Nuclear translocation of β-catenin and RUNX2 by immunofluorescent staining of HASMCs with quantitation of β-catenin and RUNX2 MFI. Bars: 20 µm. *P < 0.05; ns: not significant (n = 5).

Reciprocally, inhibition of LRP6 or DKK1 expression by siRNA abolished inhibition of calcification-induced calcium deposit in HASMCs by U0126 (Figure 4A). Correspondingly, LRP6 or DKK1 siRNA blocked the effects of U0126 on BMP2, β-catenin, RUNX2 and ALP expression (Lane 5 *vs.* 4, Figure 4B-C).

DKK1 functions in a paracrine/autocrine manner. Therefore, compared with normal IgG, neutralizing medium DKK1 protein by anti-DKK1 antibody abolished the inhibitory effects of U0126 on expression of BMP2, β-catenin, RUNX2 and ALP (Lane 5 *vs.* 4, Figure 4D). The calcification-induced expression and nuclear translocation of both β-catenin and RUNX2 were substantially attenuated by U0126 (left three panels, Figure 4E). However, anti-DKK1 antibody abrogated the function of U0126 on expression/nuclear translocation of β-catenin and RUNX2 (right two panels, Figure 4E). Correspondingly, calcification-induced expression of BMP2 and ALP was inhibited by U0126 (left halves, Figure S9), but the inhibition was attenuated by anti-DKK1 antibody (Lane 6 *vs.* 3, Figure S9).

### ERK1/2 inhibition enhances miR-126-3p maturation via AMPKα/p53 signaling pathway

**Figure 5:**
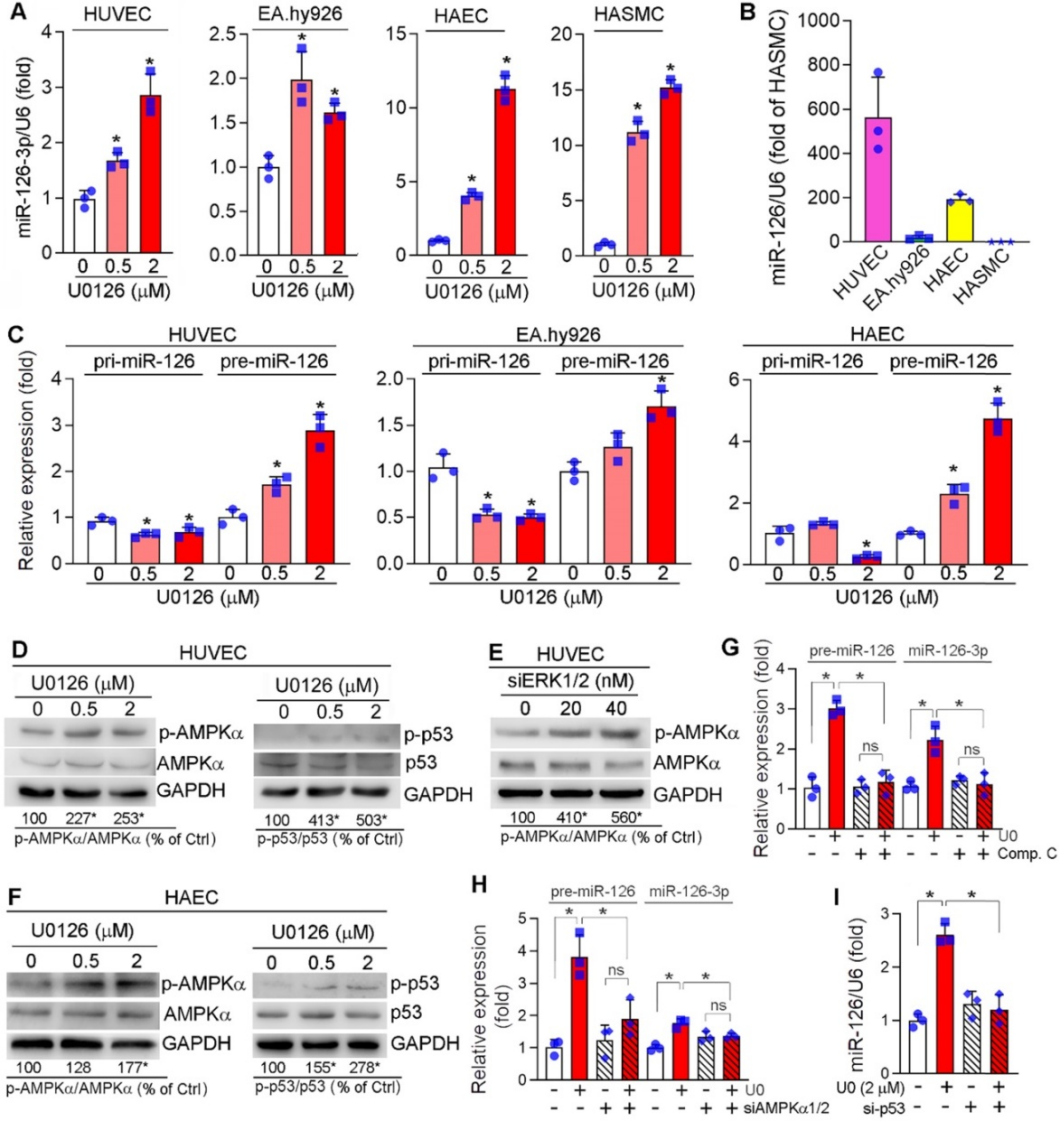
** Activation of miR-126-3p expression by ERK1/2 inhibition is mediated by activating AMPKα/p53 pathway.** (**A**) HUVECs, EA.hy926 cells, HAECs and HASMCs were treated with U0126 at the indicated concentrations overnight, followed by determination of miR-126-3p expression by quantitative miR stem-loop RT-PCR technology. *P < 0.05, (n = 3); (**B**) total RNA extracted from un-treated HUVECs, EA.hy926 cells, HAECs or HASMCs was used to determine miR-126-3p expression by quantitative miR stem-loop RT-PCR technology. (**C-D, F**) HUVECs, EA.hy926 cells or HAECs were treated with U0126 at the indicated concentrations overnight. Expression of pri-miR-126 and pre-miR-126 was determined by qRT-PCR (**C**). Expression of AMPKα, p-AMPKα, p53 and p-p53 in HUVECs (**D**) or HAECs (**F**) were determined by Western blot. *P < 0.05 *vs.* control in the corresponding group (n = 3). (**E**) HUVECs were transfected with siERK1/2 at the indicated concentration for 24 h, followed by incubation in complete medium for another 48 h. Expression of AMPKα and p-AMPKα were determined by Western blot. *P < 0.05 (n = 3). (**G**) HUVECs were treated with U0126 (2 µM) or U0126 plus Compound C (Comp. C, 10 µM) overnight followed by determination of pre-miR-126 by qRT-PCR and miR-126-3p by quantitative miR stem-loop RT-PCR technology. *P < 0.05, ns: not significant (n = 3). (**H-I**) HUVECs were transfected with control, AMPKα1/2 or p53 siRNA for 24 h. Cells were then switched to complete EC medium and cultured for another 24 h, followed by U0126 (2 µM) treatment for 24 h. Expression of pre-miR-126-3p was determined by qRT-PCR and miR-126-3p by quantitative miR stem-loop RT-PCR technology. *P < 0.05 (n = 3).

microRNAs (miRNAs), a large family of small noncoding RNAs which regulate target gene expression at the post-transcriptional level, play an important role in various diseases including cardiovascular disease. ECs and SMCs are adjacent cell types in the vasculature and ECs can produce and secret different molecules including miRNAs to influence SMC phenotype and functions. To determine if the anti-vascular calcification functions of ERK1/2 inhibition is mediated by miRNAs, we determined the changes of some key miRNAs in response to ERK1/2 inhibitor treatment in ECs, and found miR-145, miR-143, miR-155 were moderately (Figure S10A) while miR-126-3p was significantly activated by U0126 in ECs (HUVECs, EA.hy926 cells and HAECs) (Figure 5A). More importantly, U0126 also activated expression of miR-126-3p in HASMCs (Figure 5A). However, SMCs had a much lower profile of miR-126-3p than ECs (~600 fold in HUVECs, ~200 fold in HAECs, Figure 5B).

The activation of miR-126-3p expression and inhibition of SMC calcification by ERK1/2 inhibition indicates miR-126-3p could be an important mediator for ERK1/2 inhibition-reduced SMC calcification, and mainly produced by ECs. Thus, we focused on the effect of ERK1/2 inhibition on miR-126-3p expression and the underlying mechanisms in ECs. Interestingly, neither mRNA expression nor promoter activity of EGFL7, the host gene for miR-126-3p, was changed by U0126 (Figure S10B-C), indicating U0126 activated miR-126-3p in a post-transcriptional manner. Indeed, the primary transcript of miR-126 (pri-miR-126) was moderately reduced by U0126 in EA.hy926 cells, HUVECs and HAECs, while miR-126 precursor (pre-miR-126) was increased (Figure 5C), suggesting ERK1/2 inhibitor facilitate EC miR-126-3p maturation from pri-miR-126 to pre-miR-126.

Pri-miRNA is processed into pre-miRNA by Drosha complex in the nucleus. The activity of Drosha complex can be influenced by multiple interacting proteins. ERK1/2 inhibition had little effect on Drosha expression (Figure S11A). Inhibition of Drosha expression by siRNA (Figure S11B) caused accumulation of pri-miR-126 (left panel, Figure S11C) which was due to the process of pri-miRNA into pre-miRNA was reduced. In contrast, inactivation of Drosha abolished U0126-facilitated miR-126-3p maturation (middle and right panels, Figure S11C), indicating activation of miR-126-3p maturation is depended on the Drosha complex (i.e. Drosha interaction proteins) rather than expression of Drosha protein itself.

p53 can interact with the Drosha-p68 processing complex to enhance maturation of several miRNAs post-transcriptionally 47, while AMPKα can increase p53 activity 48. Previously, we reported that ERK1/2 inhibitors activate hepatic AMPKα 23. Similarly, ERK1/2 inhibitor or siRNA activated AMPKα (p-AMPKα) without affecting its expression in HUVECs (left panel of Figure 5D-E). Meanwhile, p53 phosphorylation at serine 15 (p-p53, which is mediated by p-AMPKα) was increased by U0126 (right panel of Figure 5D). Similar results were also observed in HAECs in which U0126 activated p-AMPKα and p-p53 (Figure 5F). In the presence of Compound C (an AMPKα inhibitor) or siRNA for AMPKα1/2, the induction of both pre-miR-126 and miR-126-3p by U0126 was blunted (Figure 5G-H). Inhibition of p53 expression by siRNA also blunted U0126-induced miR-126-3p expression in HUVECs (Figure 5I). Taken together, Figure 5 indicates that ERK1/2 inhibition induces miR-126-3p expression through activation of AMPKα/p53 signaling pathway.

### Activated EC miR-126-3p mediates anti-vascular calcification actions of ERK1/2 inhibition

**Figure 6:**
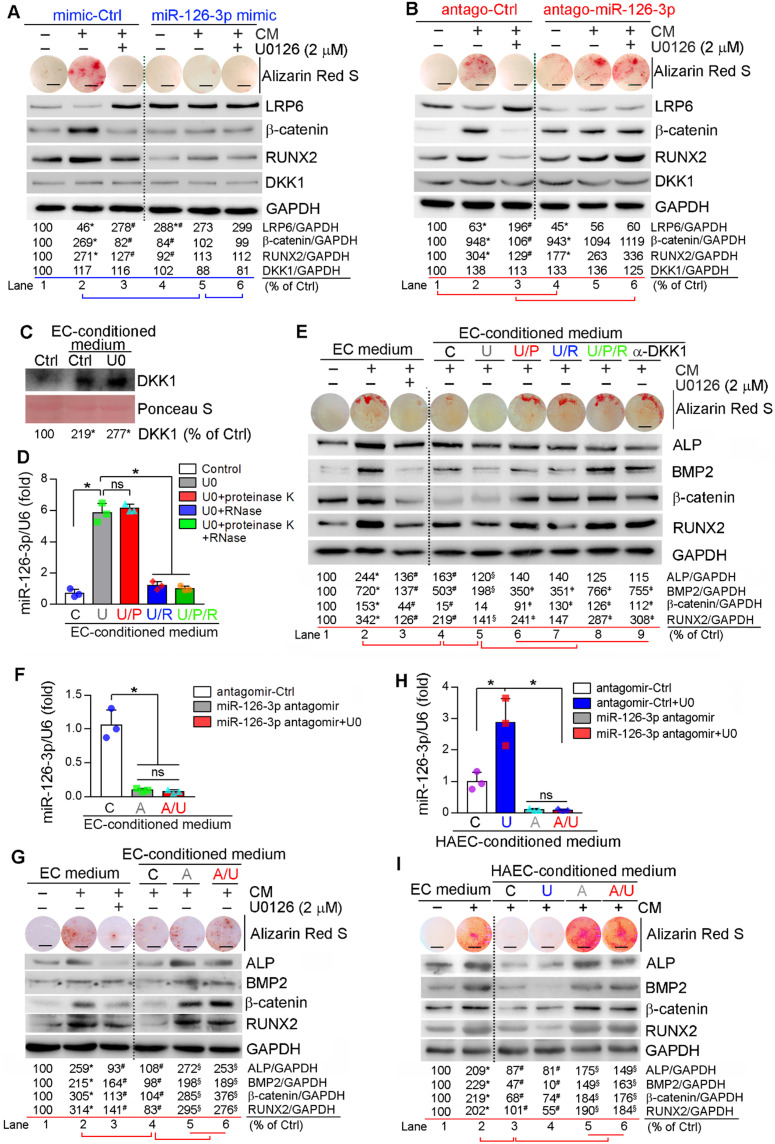
** Activated EC miR-126-3p mediates U0126-inhibited HASMC calcification.** (**A-B**) HASMCs were transfected with miR-126-3p mimic or antagomir for 24 h. Cells were then switched to complete DMEM/F12 medium, CM or CM plus U0126 (2 µM). (**C-D**) HUVECs cultured in 100-mm dishes received treatment of vehicle or U0126 (U0126-treated) at 2 µM for 24 h, followed by washing with PBS twice. Cells were continued culture in serum-free EC medium for another 24 h, and the 24-h conditioned medium from vehicle or U0126-treated HUVECs was collected separately. The vehicle-treated EC conditioned medium was named as “C” medium. The U0126-treated EC conditioned medium was named as “U” medium. HASMCs in 6-well plate was cultured with DMEM/F12 medium plus the “C” or “U” EC-conditioned medium (1 : 1) for 24 h. After washed with PBS, the cells were cultured in serum-free DMEM/F12 medium for another 24 h. Then the medium was collected and used to determine DKK1 levels by Western blot (**C**). *P < 0.05 (n = 3). The “U” medium was also divided into 4 parts, treated with PBS, proteinase K, RNase or proteinase K plus RNase, and named as “U”, “U/P”, “U/R” or “U/P/R” medium separately. After inactivation of RNase and/or proteinase K by heating, miR-126-3p levels were determined by quantitative miR stem-loop RT-PCR technology (**D**). *P < 0.05; ns: not significant (n = 3). (**E**) HASMCs were cultured in complete DMEM/F12 medium, double-concentrated CM (containing 20 mM β-glycerol phosphate and 500 µM ascorbic acid) mixed with EC medium (1 : 1), EC medium plus U0126 (2 µM) (sample #1-3), the EC-conditioned medium collected from Figure 6D (1 : 1, sample #4-8), or EC medium (1 : 1) plus anti-DKK1 antibody (sample #9). (**F, H**) HUVECs or HAEC in 60-mm dishes were transfected with control antagomir or miR-126-3p antagomir followed by U0126 (2 µM) treatment. After 24 h of transfection or plus U0126 treatment, cells were switched to serum-free EC medium and continued culture for another 24 h. The 24-h EC-conditioned medium was collected, and named as “C”, “U”, “A” and “A/U” medium, respectively, followed by determination of miR-126-3p levels by quantitative miR stem-loop RT-PCR technology. *P < 0.05; ns: not significant (n = 3). (**G**) HASMCs were cultured in complete DMEM/F12 medium, double-concentrated CM mixed with EC medium (1 : 1) or EC medium plus U0126 (2 µM) (sample #1-3); or double-concentrated CM mixed with the HUVEC-conditioned medium (1 : 1) collected from Figure 6F (1 : 1, sample #4-6). I: HASMCs were cultured in complete DMEM/F12 medium, double-concentrated CM mixed with EC medium (1 : 1) (sample #1-2); or double-concentrated CM mixed with the HAEC-conditioned medium (1 : 1) collected from Figure 6H (1 : 1, sample #3-6). (A, B, E, G, I) all the medium or plus U0126 treatment were changed daily. After 7 days of treatment, cellular calcium deposit was determined by Alizarin Red S staining. Expression of LRP6, β-catenin, RUNX2, DKK1, BMP2 and ALP in total cellular proteins were determined by Western blot. **A, B, E, G, I:** *P < 0.05 *vs.* lane 1, ^#^*P* < 0.05 *vs.* lane 2, ^§^*P* < 0.05 *vs.* lane 4; **D:** ^ǂ^*P* < 0.05 *vs.* lane 5 (n = 3). Bars: 5 mm.

Similar to *in vitro* studies, we found miR-126-3p levels in apoE^-/-^ mouse serum and aorta were increased by U0126 treatment (Figure S12), indicating miR-126-3p production and secretion are enhanced. The interactions between ECs and SMCs play important roles in different vascular biological processes including vascular homeostasis or remodeling 12, 49. Based on fact that miR-126-3p was increased by U0126 in both ECs and SMCs, while miR-126-3p levels in ECs were much higher than SMCs (Figure 5B), miR-126-3p derived from either SMCs or ECs may mediate the anti-vascular calcification functions of ERK1/2 inhibition with a much greater effect from EC miR-126-3p. To test it, we initially determined the effect of manipulated miR-126-3p on U0126-inhibited HASMC calcification. Similar to normal HASMCs, transfection of HASMCs with control mimic or antagomir did not influence CM- or U0126-regulated calcium deposit and expression of β-catenin, RUNX2 and LRP6 (left halves, Figure 6A-B). Transfection of HASMCs with miR-126-3p mimic substantially inhibited CM-induced cellular calcium deposit, associated with activation of LRP6 and inhibition of β-catenin/RUNX2 expression (lane 5 *vs.* 2, Figure 6A). No further regulation on calcium deposit or expression of β-catenin/RUNX2 by U0126 in miR-126-3p mimic-transfected HASMCs was determined (lane 6 *vs*. 5, Figure 6A). In contrast, transfection of HASMCs with miR-126-3p antagomir moderately increased cellular calcium deposit, associated with increased β-catenin/RUNX2 and decreased LRP6 expression when cells were cultured in complete DMEM/F12 medium (lane 4 *vs*. 1, Figure 6B). Although miR-126-3p antagomir had little effect on CM-induced calcium deposit and expression of β-catenin/RUNX2 (Lane 5 *vs.* 2, Figure 6B), it blocked the actions of U0126 on HASMC calcification (lane 6 *vs*. 3, Figure 6B). Therefore, Figure 6A-B confirm that miR-126-3p is an important mediator involved in U0126-inhibited HASMC calcification.

Next, we collected conditioned medium from normal or U0126-treated HUVECs. We found culture HASMCs with control EC-conditioned medium enhanced DKK1 secretion from the cells, and the secretion was further enhanced by U0126-treated EC-conditioned medium (Figure 6C). The conditioned medium collected from U0126-treated cells was further treated with proteinase K and/or RNase to inactivate miR-126-3p complex contained in the medium. Similar to serum results (Figure S12A), miR-126-3p level in EC-conditioned medium was also substantially increased by U0126 (bar “U” *vs.* “C”, Figure 6D) further confirming U0126 activates both production and secretion of miR-126-3p. Treatment of EC-conditioned medium with proteinase K had no effect on miR-126-3p stability while RNase clearly induced miR-126-3p degradation (bar “U/P”, “U/R” or “U/P/R” *vs.* “U”, Figure 6D).

HASMCs were induced calcification using a CM with doubled concentrations of β-glycerol phosphate (20 mM) and ascorbic acid (500 µM) which was further mixed with the EC-conditioned medium (1 : 1) above. Addition of normal non-cultured EC medium to CM had little effect on CM-induced calcification or anti-calcification actions of U0126 (lane 1-3, Figure 6E). In contrast, EC-conditioned medium collected from vehicle-treated HUVECs blocked calcium deposit in HASMCs and attenuated CM-induced expression of BMP2, β-catenin, ALP and RUNX2 (lane 4 *vs*. 2, Figure 6E), indicating the component(s) secreting into medium from HUVECs can inhibit vascular calcification. More reduction of cellular calcium deposit and expression of osteogenic genes in HASMCs were observed by the EC-conditioned medium collected from U0126-treated HUVECs than vehicle-treated HUVECs (lane 5 *vs*. 4**,** Figure 6E), suggesting that U0126 increases production/secretion of the anti-calcification component(s) in/from HUVECs. However, after treatment with proteinase K or RNase, the U0126-treated EC-conditioned medium had reduced anti-calcification effects (lane 6 or 7 *vs*. 5, Figure 6E), and the co-treatment of proteinase K and RNase blocked the anti-calcification actions of U0126-treated EC-conditioned medium (lane 8 *vs*. 5, Figure 6E), indicating the anti-calcification component(s) in EC-conditioned medium is incompletely inactivated by proteinase K or RNase alone, and completely inactivated by proteinase K and RNase co-treatment. Interestingly, the anti-calcification effect of EC-conditioned medium was totally abolished by anti-DKK1 antibody (lane 9 *vs*. 5, Figure 6E), indicating DKK1 is a downstream target of the EC-conditioned medium.

To further identify the main anti-calcification component in EC-conditioned medium in response to U0126 treatment, we transfected HUVECs and HAECs with control antagomir or miR-126-3p antagomir plus U0126 treatment. Figure 6F, 6H shows that transfection of miR-126-3p antagomir blocked miR-126-3p secretion from cells regardless of U0126 treatment. We collected the above EC-conditioned medium and named as “C”, “U”, “A” or “A/U” EC-conditioned medium, separately. The medium was mixed with a CM having doubled concentrations of β-glycerol phosphate and ascorbic acid (1 : 1) and then used to induce HASMC calcification. Compared to normal EC medium, the “C” or “U” EC-conditioned medium inhibited CM-induced cellular calcium deposit and expression of BMP2, β-catenin, ALP and RUNX2 in HASMCs (lane 4 *vs*. 2, Figure 6G; lane 3 or 4 *vs.* 2, Figure 6I). However, the “A” and “A/U” EC-conditioned medium which contained little miR-126-3p (Figure 6F, 6H) had little effect on CM-induced calcification or expression of osteogenic molecules (lane 5 and 6 *vs*. 4, Figure 6G, 6I).

Finally, we collected the conditioned medium from ECs co-transfected with control siRNA plus control antagomir or miR-126-3p antagomir, ERK1/2 siRNA plus control antagomir or miR-126-3p antagomir, separately. Similar to ERK1/2 inhibitor (Figure 6D), ERK1/2 siRNA also significantly increased miR-126-3p levels in the medium, but the increase was blocked by transfection of miR-126-3p antagomir (Figure S13A). The conditioned medium collected from the transfected ECs above was named as “C” (siCtrl + Ctrl antagomir), “SI” (siERK1/2 + Ctrl antagomir), “A” (siCtrl + miR-126-3p antagomir) or “A/SI” (miR-126-3p antagomir + siERK1/2) medium, mixed with a CM having doubled concentrations of β-glycerol phosphate and ascorbic acid (1 : 1), and used to culture HASMCs, separately. After calcification induction, we found both “C” and “SI” medium inhibited CM-induced calcium deposit, reduced CM-activated expression of the pro-calcification molecules (ALP, BMP2, RUNX2 and β-catenin) while activated CM inhibited expression of the anti-calcification molecule, LRP6 (lane 3, 4 *vs*. 2, Figure S13B). The greater effect was observed by “SI” medium than “C” medium (lane 4 *vs*. 3, Figure S13B) which could be due to more miR-126-3p contained in “SI” medium than “C” medium (Figure S13A). Interestingly, “A” or “A/SI” medium had slight effect on CM-induced calcium deposit or expression of calcification-related molecules (lane 5 and 6 *vs.* 2, Figure S13B-C). Taken together, Figure 6 and Figure S13 suggest that activated miR-126-3p production, particularly from ECs, is the important mediator for anti-vascular calcification actions of ERK1/2 inhibition.

### Activated EC miR-126-3p inhibits adhesion of monocytes to ECs

EC dysfunction also accelerates atherosclerosis and vascular calcification. Previous studies have demonstrated miR-126-3p also inhibits adhesion of leukocytes to ECs by reducing vascular cell adhesion molecule-1 (VCAM-1) 19. Activated VCAM-1 in ECs can facilitate monocyte adhesion to endothelium. Reduction of VCAM-1 expression in arterial wall of apoE^-/-^ mice by U0126 (Figure S14A) indicates that U0126 may reduce adhesion of monocytes to ECs. Indeed, *in vitro*, treatment of LPS-activated HUVECs with U0126 or PD98059 decreased the adhesion of THP-1 cells, a human monocytic cell line (Figure S14B). miR-126-3p mimic reduced while miR-126-3p antagomir increased adhesion of THP-1 cells to HUVECs. More importantly, both miR-126-3p mimic and antagomir abolished PD98059 or U0126-reduced adhesion of THP-1 cells to HUVECs (Figure S14B), which suggests that ERK1/2 inhibition reduced adhesion of monocytes to ECs in a miR-126-3p-dependent manner.

## Discussion

Vascular calcification plays an important role in cardiovascular diseases, such as atherosclerosis, restenosis and rupture of vulnerable plaques. Atherosclerotic calcification is expected to have complex effects on plaque vulnerability, which depends on the type of calcification. Generally, the spotty calcification is considered as a more potent risk factor of plaque rupture compared to the large calcification 3, 4, 50. While the medial arterial calcification reduces elasticity and compliance of the vessel wall, and has been recognized as a well-known predictive risk factor of subsequent cardiovascular mortality 51. Multiple factors are involved in vascular calcification 1, 52. Our previous study demonstrates that ERK1/2 inhibition not only blocks LXR-activated lipogenesis but also synergizes LXR-inhibited atherosclerosis. ERK1/2 inhibition can reduce macrophage/foam cell formation and activate reverse cholesterol transport 23, 29, 39. Indeed, previous studies have discovered that ERK1/2 regulates SMC osteogenesis by multiple mechanisms including the direct phosphorylation of RUNX2, activation of CK2α-dependent α-catenin phosphorylation for release of β-catenin 26-28.

Herein, we initially observed the activated ERK1/2 was positively correlated to human aortic valves calcification (Figure 1A-B), indicating ERK1/2 signaling may be also involved in the pathogenesis of vascular calcification owing to the similar pathological process between aortic valve and vascular calcification. Indeed, we determined the protective role of ERK1/2 inhibition against atherosclerotic calcification and medial arterial calcification. The protections against vascular calcification were confirmed by the actions of ERK1/2 inhibition *in vivo*, *ex vivo* and *in vitro*. *In vivo*, U0126 reduced calcium deposit in lesion areas and aortas from proatherogenic and medial arterial calcification mice (Figure 2A-E). The substantial reduction of calcium deposit in aortic rings or SMCs induced by calcification-conditioned medium *ex vivo* or *in vitro* was observed by ERK1/2 inhibitor and siRNA (Figure 2F, H, J; S3), and expression of osteogenic genes (Figure 2G, I, K; S4A). Associated with regulation of SMC calcification, ERK1/2 activity was concomitantly changed (Figure 2G, I).

Recently, Ricard *et al.* reported that endothelial ERK2 knockout in adult ERK1^-/-^ mice resulted in a rapid onset of hypertension and a decrease in eNOS expression 24. All the mice died within 5 weeks owing to widespread endothelial-to-mesenchymal transition (EndMT). Moreover, nicotine promotes atherosclerosis by enhancing EndMT *via* activation of ERK1/2. Blocking ERK1/2 with inhibitor efficiently preserves endothelial phenotype upon nicotine stimulation 53. However, in our study we determined U0126 had little effect on cell apoptosis both *in vitro* and *in vivo* (Figure S6, S7). Moreover, in the aortic plaques, U0126 increased SMC proliferation, another mechanism for ERK1/2 inhibition-increased lesion stability. We believed that U0126 at the dose used in this study can decrease the abnormal elevation of ERK1/2 activity to the basal level (Figure 2G, I) in the context of atherosclerosis progression, while still preserved the physiological function of ERK1/2 in artery wall 23.

The anti-vascular calcification properties of ERK1/2 inhibition were correlated to activation of DKK1 secretion and LRP6 expression (Figure 3A-C, G; S8A-C), thereby inactivating canonical and non-canonical Wnt pathways to reduce β-catenin expression/nuclear translocation (Figure 3D-F, I; S8A, S8E). Although DKK1 is expressed by SMCs, it interacts with LRP5/6 extracellularly to inactivate Wnt signaling, indicating a paracrine/autocrine manner involved 54. Therefore, either inhibition of DKK1 expression by siRNA or neutralization of DKK1 in medium by anti-DKK1 antibody blocked the anti-calcification actions of U0126 (Figure 4A, C-E; S9). Moreover, EC-conditioned medium can increase SMC DKK1 secretion (Figure 6C), and the anti-calcification function of EC-conditioned medium was abolished by anti-DKK1 antibody (Figure 6E), suggesting the EC-conditioned medium-induced DKK1 secretion could be a downstream effector of miR-126-3p.

Meanwhile, LRP6 expression in SMCs was activated by ERK1/2 inhibition, miR-126-3p mimic or EC-conditioned medium also enhanced SMC LRP6 expression (Figure 3G, I; 6A; S13B-C) and inhibited by miR-126-3p antagomir (Figure 6B), suggesting LRP6 is another downstream effector of miR-126-3p. Although LRP6 is one of Wnt coreceptors, it can inactivate non-canonical Wnt pathway. Lack of LRP6 expression in SMC increases expression of Frizzled 10 and Wnt ligands, such as Wnt7b/10a, thereby activating non-canonical signaling and aortic calcification 46. Consistently, we found SMC calcification inhibited LRP6 while ERK1/2 inhibition activated LRP6 expression (Figure 3G, I). Reciprocally, LRP6 siRNA blocked the effects of U0126 on calcification and expression of osteogenic genes (Figure 4A-B). Similar to vascular calcification, compared to the adjacent tissue, we found increased BMP2/RUNX2 and reduced DKK1 in the calcification part of human calcific aortic valve (Figure 1D). Interestingly, we found either siDKK1 or siLRP6 can abolish U0126-inhibited calcification (Figure 4A), thus, we speculate that the interaction between DKK1 and LRP6 may be essential for ERK1/2 inhibitor-reduced both non-canonical and canonical Wnt signaling pathway. In the canonical Wnt signaling pathway, DKK1 inhibited β-catenin nuclear translocation by interacting with LRP5/6. Therefore, inactivation either DKK1 or LRP6 can abolish U0126-inhibited canonical Wnt signaling pathway. Reciprocally, previous study indicates LRP6 involved in the non-canonical Wnt signaling pathway. Our results suggest DKK1 may also be involved in the non-canonical Wnt signaling pathway which may need more investigation in the future.

Several miRNAs display different functions in vascular biology by various mechanisms 55-58. For instance, miR-155 enhances lesion development by activating macrophage chemokine CCL2 expression, which promotes the recruitment of monocytes to atherosclerotic plaques, and inhibits BCL6 expression, a transcription factor reducing NF-κB expression. In contrast, activation of miR-126-3p expression inhibits atherosclerosis by activating CXCL12/CXCR4 pathway through inactivation of RGS16 in ECs 18. The TNF-α-activated VCAM-1 in human aortic ECs (HAECs) is enhanced by postprandial triglyceride-rich lipoproteins (TGRL) isolated from proatherogenic subjects, but reduced by TGRL isolated from antiatherogenic subjects. Interestingly, miR-126-3p expression in HAECs is inversely regulated by TGRL, while inhibition of miR-126-3p expression enhances TNF-α-activated VCAM-1 expression 59. Herein, we defined the important role of miR-126-3p in ERK1/2 inhibition-reduced vascular calcification. The protection of ERK1/2 inhibition was linked to activation of miR-126-3p expression mainly in ECs in an AMPKα/p53-dependent pathway (Figure 5, 6; Figure S10, S11).

The interactions between ECs and SMCs greatly influence vascular homeostasis including vascular calcification. For instance, treatment of ECs with TNF-α activates release of endothelial microparticles (EMPs) which contain activated BMP2. Therefore, EMPs isolated from TNF-α-treated EC-conditioned medium enhances SMC calcification 12. We determined U0126 increased circulating and aortic miR-126-3p levels (Figure S12). ERK1/2 inhibition activated miR-126-3p expression in both ECs and SMCs (Figure 5A) and manipulation of miR-126-3p levels in SMCs influenced U0126-inhibited SMC calcification (Figure 6A-B). We observed ERK1/2 inhibition increased miR-126-3p levels in circulation and EC-conditioned medium (Figure S12A, S13A; 6D, H), suggesting ERK1/2 inhibition activates both miR-126-3p expression and secretion. We further determined that ERK1/2 inhibition-treated EC-conditioned medium potently reduced SMC calcification in a miR-126-3p-dependent manner (Figure 6D-I; S13B-C), suggesting activated miR-126-3p is the mediator for anti-vascular calcification actions of ERK1/2 inhibition. Interestingly, treatment of EC-conditioned medium with proteinase K had no effect on miR-126-3p stability (Figure 6D). However, it can partially reduce the anti-calcification effects of U0126-treated EC-conditioned medium (Figure 6E). We speculate that the results may be caused by the following reasons: 1) miR-126-3p was secreted by apoptotic body or combining with Argonaute2 (Ago2) 18, 60. Since U0126 had no effect on cell viability and apoptosis (Figure S6, S7), the U0126-increased miR-126-3p secretion may rely on Ago2. Although proteinase K had no effect on miR-126-3p degradation, it can degrade Ago2, which may affect miR-126-3p uptake by target cells; 2) DKK1 may also be produced by ECs and secret into medium, which can be degraded by proteinase K.

In the present study, we show that ERK1/2 inhibition reduced vascular calcification. Although U0126 was not moved to clinical application due to its pharmaceutical limitations, it has demonstrated to be an invaluable academic research tool to investigate the role of ERK1/2 pathway in normal cell physiology and disease. In this study, we used it at a very low dose and observed no noticeable side effects. Our study indicates ERK1/2 inhibitors, some of them are currently in clinical trials for cancer treatment, may also have potential clinical benefits for calcification-related vascular diseases, such as atherosclerosis and medial arterial calcification. Mechanistically, we determined that miR-126-3p can influence canonical and non-canonical Wnt pathways by promoting LRP6 and DKK1 expression/secretion. However, the sequence alignment assay for miRNA target genes by the online miRNA target scanning tools (miRbase, miRtarbase and TargetScan) indicates LRP6 but not DKK1 may be the target gene of miR-126-3p. Treatment of VSMCs with EC-derived microparticles (EMPs) which are generated from the apoptotic ECs and rich in miR-126-3p reduces SMC proliferation, and the reduction is related to simultaneous inhibition of SMC LRP6 and β-catenin expression 61. However, another study demonstrates that treatment of VSMCs with EC-conditioned medium directly increases SMC proliferation in miR-126-3p-depednet manner and related to inhibition of other targets, not LRP6 60. Therefore, the effect of miR-126-3p on VSMC phenotype is controversial, and related to the microenvironment of miR-126-3p presents. In our study, we found that treatment of SMCs in calcification condition with miR-126-3p either directly or in EC-conditioned medium potently inhibits SMC osteogenesis/calcification by inhibiting expression of osteogenic genes including β-catenin expression through activation of LRP6 and DKK1 expression (Figure 6, S13), indicating the effect of miR-126-3p on SMCs is greatly influenced by the microenvironment. In addition, the duration of treatment in our study is 7 days which is much longer than EMP treatment, which may make contribution to the different effect of miR-126-3p on SMC. We will complete more investigation in the future to further unveil the mechanisms of miR-126-3p on SMC as well as its other potential target(s).

Macrophage also plays an important role in calcification. Aikawa *et al.* showed that macrophage infiltration and inflammation precede both the osteogenic conversion of VSMCs and the vesicle release associated with the generation of the first nidus of microcalcification, indicating inflammation is a potent initiator of calcification in atherosclerosis 62. Macrophages release calcifying matrix vesicles enriched in S100A9 and annexin V, which contribute to accelerated microcalcification 63. In addition, oxidized lipoproteins stimulate vascular calcification by driving osteoblastic differentiation of VSMCs while inhibiting osteoclast differentiation of macrophages *via* foam cell formation 64. Previously, we found MEK1/2 inhibitors also induce reverse cholesterol transport and inhibit macrophage/foam cell formation by activating macrophage ABCA1/G1 expression 29. Moreover, we determined U0126 inhibited VCAM-1 expression, thereby reducing adhesion of monocytes to ECs in a miR-126-3p dependent manner (Figure S14). The reduced monocyte adhesion can result in reduced macrophages differentiation and accumulation within plaques which also contributed to U0126-inhibited calcification. It may also suggest that miR-126-3p functions as an anti-inflammatory mediator of ECs in atherosclerosis as well as vascular calcification, which needs more investigation to be pursued in the future.

In conclusion, our study demonstrates that ERK1/2 inhibition reduces atherosclerosis and vascular calcification. In addition to improvement of macrophage cholesterol metabolism, protection against vascular calcification is another important anti-atherogenic property of ERK1/2 inhibition. Mechanistically, activated miR-126-3p expression and secretion from ECs enhances SMC DKK1/LRP6 secretion/expression which inactivates both non-canonical and canonical Wnt signaling pathway. miR-126-3p is an important mediator for anti-atherogenic properties of ERK1/2 inhibition and interactions between ECs and SMCs (Graphical abstract).

## Supplementary Material

Supplementary figures and tables.

## Abbreviations

ALP: alkaline phosphatase
AMPK: AMP-activated protein kinase
BMP2: bone morphogenetic protein 2
CAVD: calcific aortic valve disease
CKD: chronic kidney disease
CM: calcification medium
DKK1: dickkopf 1
EGFL7: epidermal growth factor like domain multiple 7
ERK1/2: extracellular signal-regulated kinase 1 and 2
HFD: high-fat diet
HUVEC: human umbilical vein endothelial cell
HAEC: human aortic endothelial cell
LRP5/6: LDL receptor related protein 5/6
LXR: liver x receptor
MEK1/2: mitogen-activated protein kinase kinase 1 and 2
OPN: osteopontin
pre-miR-126: miR-126 precursor
pri-miR-126: primary transcript of miR-126
RUNX2: Runt related transcription factor 2
SMC: smooth muscle cell
USF1: upstream stimulatory factor 1
VCAM-1: vascular cell adhesion molecule-1
VD_3_: Vitamin D_3_
