# Supplementary material for: ERK1/2 inhibition reduces vascular calcification by activating miR-126-3p-DKK1/LRP6 pathway
Source: Theranostics. 2021 Jan 1;11(3):1129–46. doi: 10.7150/thno.49771 (PMC7738895; doi:10.7150/thno.49771)
Supplement: Supplementary file 1 — Supplementary figures and tables. [file thnov11p1129s1.pdf]

Supplementary materials
for

ERK1/2 inhibition reduces vascular calcification by activating miR-126-3p-DKK1/LRP6 pathway

Peng Zeng[1*], Jie Yang[1*], Lipei Liu[1], Xiaoxiao Yang[2], Zhi Yao[3], Chuanrui Ma[4], Haibo Zhu[5], Jiamin Su[2], Qian Zhao[2], Ke Feng[1], Shu Yang[1], Yan Zhu[6], Xiaoju Li[1], Wenguang Wang[7], Yajun Duan[2], Jihong Han[1,2,†], Yuanli Chen[2,†]

[1]College of Life Sciences, State Key Laboratory of Medicinal Chemical Biology, Key Laboratory of Bioactive Materials of Ministry of Education, Nankai University, Tianjin, China; [2]Key Laboratory of Metabolism and Regulation for Major Diseases of Anhui Higher Education Institutes, Hefei University of Technology, Hefei, China; [3]Tianjin Medical University, Tianjin, China; [4]First Teaching Hospital of Tianjin University of Traditional Chinese Medicine, Tianjin, China; [5]Chinese Academy of Medical Sciences and Peking Union Medical College, Beijing, China; [6]Tianjin University of Traditional Chinese Medicine, Tianjin, China; [7]Tianjin Chest Hospital, Tianjin, China

I. Supplementary methods

Reagents

Rabbit anti-AMPKα (Cat#: sc-25792), phosphorylated AMPKα (p-AMPKα, Cat#: sc-101630), phosphorylated p53 at S15 [p-p53 (S15), Cat#: sc-101671], rat anti-MOMA2 (Cat#: sc-59332) and mouse anti-LRP6 (Cat#: sc-25317), SMA (Cat#: sc-130617) monoclonal antibodies were purchased from Santa Cruz Biotechnology (Dallas, Texas, USA). Rabbit anti-ALP (Cat#: 11187-1-AP), BMP2 (Cat#: 18933-1-AP), β-catenin (Cat#: 51067-2-AP), GAPDH (Cat#: 10494-1-AP), Lamin A/C (Cat#: 10298-1-AP), OPN (Cat#: 22952-1-AP), RUNX2 (Cat#: 20700-1-AP) and VCAM-1 (Cat#: 11444-1-AP) polyclonal antibodies, mouse anti-CD31 (Cat#: 66065-1-IG) monoclonal antibody, goat anti-rabbit IgG (H+L)-TRITC (Cat#: SA00007-2) and goat anti-rabbit IgG (H+L)-FITC (Cat#: SA00003-2) antibodies were purchased from Proteintech Group (Chicago, IL, USA). Rabbit anti-p53 (Cat#: 9282S), ERK1/2 (Cat#: 9102S) and phosphorylated ERK1/2 (p-ERK1/2, Cat#: 9101S) polyclonal antibodies were purchased from Cell Signaling Technology (Danvers, MA, USA). Rabbit anti-DKK1 (Cat#: NBP2-24701) and Drosha (Cat#: NBP1-03349) polyclonal antibodies were purchased from Novus Biologicals (Littleton, CO, USA). Rabbit anti-Ki-67 (Cat#: ab16667) polyclonal antibody was purchased from Abcam (Cambridge, MA, USA). Rabbit anti-JNK1/2/3 (Cat#: A4867), phosphorylated JNK1/2/3 (p-JNK1/2/3, Cat#: AP0631), p38 MAPK (Cat#: A14401) and phosphorylated p38 MAPK (p-p38 MAPK, Cat#: AP0526) polyclonal antibodies were purchased from ABclonal (Wuhan, China). Mouse anti-RUNX2 monoclonal antibody (Cat#: D130-3) was purchased from MBL CO., LTD. (Chiba, Japan). Goat anti-mouse IgG (whole molecule)-FITC antibody (Cat#: F0257) and goat anti-mouse IgG (whole molecule)-TRITC antibody (Cat#: T5393) were purchased from Sigma-Aldrich (St Louis, MO, USA). miR-126-3p antagomir (Cat#: miR30000445-1-2), miR-126-3p mimic (Cat#: miR10000445-1-2), siRNA against human ERK1/2 (Cat#: siB13329174717-1-5) AMPKα1 (Cat#: siG000005562A-1-5) and AMPKα2 (Cat#: siG000005563A-1-5) were purchased from Guangzhou Ribobio (Guangzhou, Guangdong, China). siRNAs against human p53 (Cat#: sc-29435), DKK1 (Cat#: sc-37082), LRP6 (Cat#: sc-37233) and Drosha (Cat#: sc-44080) were purchased from Santa Cruz Biotechnology. U0126 (Cat#: U6770) and PD98059 (Cat#: P4313) were purchased from LC Laboratories (Woburn, MA, USA).

Preparation of plasmid DNA and determination of EGFL7 promoter activity

The human EGFL7 promoter (from -1102 to +192) was constructed by PCR with human genomic DNA isolated from HUVECs and the following primers: forward, 5’-AGGGTACCCCCACAACTCCTCTCCAACC-3’; and backward, 5’-AACCTCGAGGTCGCGCTGCCTCGT-3’. After the sequence was confirmed, the PCR product was digested with KpnI and XhoI, followed by

ligation into pGL4.10 luciferase reporter vector (Promega, Madison, WI, USA). To analyze promoter activity, EA.hy926 cells were cultured in 48-well plates and transfected with the DNA for EGFL7 promoter and *Renilla* luciferase (for internal normalization) using Lipofectamine 2000 reagent. After 24 h of transfection or plus treatment, cells were lysed and the cellular lysate was determined activity of *Firefly* and *Renilla* luciferases using the Dual-Luciferase Reporter Assay System (Promega).

Determination of calcification, expression of calcification-related genes in human calcific aortic valve samples

The study with human samples was approved by the Ethical Review Board of Tianjin Chest Hospital (Tianjin, China) and adhered strictly to the Declaration of Helsinki Principle 2008. All samples were collected after the informed written consents were signed by patients and their family members.

Transthoracic echocardiography diagnostic criteria for aortic valve calcification was leaf thickness > 2 mm and local echo enhancement in aortic valve [1]. Dual source computed tomography (DSCT) diagnostic criteria was that lesions were founded in aortic valves or in aortic root and the density was more than 130 HUs in 3 or more consecutive pixel [2]. The double positive patients were assigned as calcific aortic valve disease (CAVD). Inclusion criteria: more than 60 years old and echocardiography and cardiac DSCT were defined as CAVD patients, who had to receive the valve resection operation. Exclusion criteria included rheumatic valvar heart disease, congenital aortic valve malformation, Marfan syndrome, infected endocarditis, parathyroid disease and renal failure. Finally, 15 patients with CAVD and undergoing surgical resection of the aortic valve were enrolled in this study in Tianjin Chest Hospital, China. All researchers were blinded to the grouping.

The resected valve sample was further divided into two parts: calcification area which was confirmed by von Kossa staining (white and firm tissues, Figure 1A) and adjacent area (brownness and soft tissues, Figure 1A). Total RNA was extracted separately from these two parts and used to determine expression of BMP2, RUNX2, DKK1 and LRP6 by qRT-PCR. Part of the valve sample was removed and fixed in 10% neutral formalin, dehydrated and embedded in paraffin. The 5-μm paraffin sections were prepared and used to detect vascular calcification by Alizarin Red S staining. Expression of p-ERK1/2 and RUNX2 were determined by immunofluorescent staining [3]. After quantitation, the correlation coefficient between RUNX2 MFI and p-ERK1/2 MFI was calculated using Pearson's correlation test and one-tailed p-value was calculated for 95% confidence interval.

Quantitative real time RT-PCR (qRT-PCR)

After treatment, total RNA was extracted from tissue samples or cells using Trizol reagent (Invitrogen, Carlsbad, CA, USA). The same amount of total RNA from each sample was used to conduct reverse transcription (RT) using a

reverse transcription kit (New England Biolabs, Ipswich, USA) with oligo(dT)$_{15}$ primer. The qPCR reaction was performed using SYBR Green PCR Master Mix (Vazyme, Nanjing, Jiangsu, China) and the primers with sequences listed in Table S1. Expression of EGFL7, p53, AMPKα1 and AMPKα2 mRNA in HUVECs; USF1, RUNX2, Osx, SOX9, DKK1, LRP5 and LRP6 mRNA in HASMCs were normalized by glyceraldehyde-3-phosphate dehydrogenase (GAPDH) mRNA in the corresponding samples. Levels of miR-126-3p in serum, tissue, cells or EC-conditioned medium, miR-145, miR-143 and miR-155 in cells were analyzed by quantitative miR stem-loop RT-PCR technology (Ambion) [4].

The relative amount of pri-miR-126 and pre-miR-126 was determined using the method described by Schmittgen [5]. Briefly, cDNA was generated by reverse transfection with pre-miR-126 backward primer. Two panels of qPCR were completed in parallel: **1)** with pri-miR-126 forward/backward primers and the C_T value was defined as $C_{T\text{pri-miR-126}}$; **2)** with pre-miR-126 forward/backward primers and the C_T value was defined as $C_{T\text{precursors of miR-126}}$ (precursors of miR-126 is defined as the sum of pri-miR-126 and pre-miR-126). The pri-miR-126 and/or pre-miR-126 were calculated based on the following equations: the pri-miR-126 = $2^{-\triangle C_{T1}}$, where $\triangle C_{T1} = (C_{T\text{pri-miR-126}} - C_{T\text{GAPDH}})$; the sum of pri-miR-126 and pre-miR-126 = $2^{-\triangle C_{T2}}$, where $\triangle C_{T2} = (C_{T\text{precursors of miR-126}} - C_{T\text{GAPDH}})$; then, the pre-miR-126 = $2^{-\triangle C_{T2}} - 2^{-\triangle C_{T1}}$. Finally, we normalized the qRT-PCR results of each sample to the mean of control value.

Transfection of siRNA, miR-126-3p mimic or antagomir

HUVECs, HAECs and HASMCs in 6-well plates (for Western blot or qRT-PCR) or 24-well plates (for Alizarin Red S staining) were transfected with siRNA, miR-126-3p mimic or antagomir using Lipofectamine® RNAiMAX Transfection Reagent (Invitrogen). For Western blot and qRT-PCR analysis, after 24 h of transfection HUVECs were switched into complete EC medium and cultured for another 24 h, then received treatment in serum-free medium for 18 h.

HASMCs were induced calcification by culture in complete DMEM/F12 (1 : 1) medium containing 10 mM β-glycerol phosphate and 250 μM ascorbic acid (it was named as "CM") or CM plus siRNA transfection for 7 days. The transfection was repeated on day 3 of calcification induction. At the end of treatment, HASMCs were used to determine calcification using Alizarin Red S staining or protein expression by Western blot or immunofluorescent staining.

Determination of calcification

Atherosclerotic calcification in aortic root cross sections and medial arterial calcification in thoracic aorta cross sections of Vitamin D_3 (VD_3)-induced medial arterial calcification model were determined by Alizarin Red S and von Kossa staining [6], respectively. Briefly, sections were washed twice with PBS and incubated in 1% Alizarin Red S solution (pH 4.2) for 30 min at room

temperature, followed by rinsing twice with PBS. The von Kossa staining was completed as follows: sections were treated with 5% silver nitrate solution under ultraviolet light for 40 min, then washed with deionized water for 3 times and once with 5% sodium thiosulfate, followed by counterstaining with fast red solution for nuclei. All the images were captured with a Leica DM5000B microscope (Wetzlar, Germany). For whole-mount artery staining, mouse aortic tissue was fixed in 95% ethanol for over 24 h, and then stained with 0.003% Alizarin Red S in 1% potassium hydroxide for 30 h. Aortas were then washed twice with 2% potassium hydroxide and photographed with an inverted microscope.

To quantitatively determine calcium content, aortic samples were homogenized in 0.1 N HCl. A portion of homogenate was centrifuged for 10 min at 13,000 rpm, and the supernatant was used to determine calcium content using the Calcium LiquiColor kit (Biovision, Inc, San Francisco, USA), based on instructions from the manufacturer. The rest of homogenate was used to determine the dry weight of aortas to normalize calcium content in aortas, which was expressed as μg/mg of aortic dry weight.

In vitro, the effect of ERK1/2 inhibition on HASMC calcification was determined as follows: HASMCs in 24-well plates were cultured in complete DMEM/F12 medium (1 : 1) (normal medium for SMC culture), CM or CM plus indicated treatment for 7 days. The medium was changed every 2 days. At the end of treatment, cells were conducted Alizarin Red S staining to determine calcium deposition, and the images of cells were captured using a Leica microscope. Cells were also used to extract total cellular calcium followed by calcium quantitative assay with the Calcium LiquiColor kit, and normalization by cellular proteins. The cellular calcium content was expressed as μg/mg protein.

The effect of ERK1/2 inhibition on vascular calcification was also determined with aortic rings *ex vivo*. ApoE$^{-/-}$ mice (~8-week old) were anesthetized and euthanized in a CO_2 chamber, followed by collection of thoracic aortas. After removal of adjacent tissues carefully under a dissection microscope, thoracic aortas were cut into 5 mm long each and cultured in normal DMEM/F12 medium, CM or CM plus U0126 treatment. All the medium were changed once every two days. After culture for 2 weeks, the rings were fixed in 4% paraformaldehyde/PBS overnight, followed by preparation of 5-μm frozen sections and conduction of Alizarin Red S and von Kossa staining.

Immunofluorescent staining

Expression of BMP2, RUNX2, DKK1, LRP6, β-catenin, OPN, Ki-67 and VCAM-1 in aortic root, BMP2 and RUNX2 in thoracic artery were determined by immunofluorescent staining of the 5-μm frozen sections of the corresponding samples and primary antibodies as described [7].

Expression of β-catenin, RUNX2, BMP2 and ALP in HASMCs were also determined by immunofluorescent staining. Briefly, cells were plated on coated

glass slides in 20-mm dishes and cultured in the corresponding medium. At ~90% confluence, cells received the indicated treatment. After removal of the treatment medium from dishes, cells were fixed in 4% PFA for 20 min, washed twice with PBS for 10 min, and blocked with 2% BSA for 2 h at room temperature. Cells were then incubated with primary antibody overnight at 4 °C followed by incubation with FITC-conjugated goat anti-rabbit IgG for 2 h at room temperature. Cells were then stained with DAPI solution to detect nucleus. The slides were removed from dishes followed by observation and photographed with a fluorescence microscope (Leica). The mean fluorescent intensity (MFI) of immunofluorescent image was calculated as described [8].

The frozen sections or cells were also used to conduct negative control immunofluorescent staining using normal IgG to replace primary antibody.

Western blot

Expression of target proteins were determined by Western blot with total cellular proteins, concentrated conditioned medium or nuclear proteins was determined by Western blot. After treatment, conditioned medium was collected and concentrated. The cells remaining in dishes were washed with PBS and then lysed in an ice-cold lysis buffer (50 mM Tris, pH 7.5, 150 mM NaCl, 1% Triton X-100, 1% sodium deoxycholate, 1 mM PMSF, 50 mM sodium fluoride, 1 mM sodium orthovanadate, 50 μg/mL aprotinin/leupeptin). The cellular lysate was centrifuged and the supernatant was retained as total cellular proteins. Nuclear proteins were extracted from HASMCs using a commercial kit purchased from Solarbio (Beijing, China). The means of statistical analysis of three independent experiments were shown below the representative images, and the dot-like plots were shown in Figure S16.

Collection and treatment of the EC-conditioned medium

HUVECs in 100-mm dishes were treated with vehicle or 2 μM U0126 for 24 h. After treatment, cells were washed twice with PBS, and then cultured in serum-free EC medium for 24 h. The 24-h EC-conditioned medium from each group was collected individually. The EC-conditioned medium collected from vehicle-treated or control HUVECs was named as “C” medium. The EC-conditioned medium collected from U0126-treated HUVECs was divided into 4 parts and received the following treatment: PBS, proteinase K (20 μg/mL), RNase (50 μg/mL), and proteinase K plus RNase, respectively. After addition of enzyme(s), the medium was incubated for 1 h at 37 °C to dysfunction the secreted miRNA complex. After reaction, the added enzyme(s) was inactivated by heating for 10 min at 95 °C. The above EC-conditioned medium after enzyme(s) treatment was named as “U”, “U/P”, “U/R” and “U/P/R” medium, respectively. After determination of miR-126-3p levels, these EC-conditioned medium were used for experiment in Figure 6D-E.

To verify if the inhibition of HASMC calcification by EC-conditioned medium is due to secreted miR-126-3p, HUVECs or HAECs at the same

confluence were transfected with control antagomir or miR-126-3p antagomir for 24 h. After transfection, cells were switched into complete EC medium and continued culture for another 24 h, followed by cultured in serum-free EC medium or plus U0126 (2 μM) treatment for 24 h. After treatment, cells were washed twice with PBS, and then cultured in serum-free EC medium for another 24 h. The 24-h EC-conditioned medium in the last step was collected, and named as "C" (cells transfected with control antagomir), "U" (cells transfected with control antagomir plus U0126 treatment), "A" (cells transfected with miR-126-3p antagomir) or "A/U" (cells transfected with miR-126-3p antagomir plus U0126 treatment) medium. After determination of miR-126-3p levels, these EC-conditioned medium were used for experiment in Figure 6G or 6I, respectively.

To further verify if ERK1/2 inhibition by siRNA can also affect miR-126-3p secretion as well as the anti-calcification effect of the EC-conditioned medium, HUVECs in 4 dishes at the same confluence were transfected with control antagomir plus control siRNA, control antagomir plus ERK1/2 siRNA, miR-126-3p antagomir plus control siRNA, or miR-126-3p antagomir plus ERK1/2 siRNA for 24 h. After transfection, cells were switched into complete EC medium and continued culture for another 24 h. Then, cells were washed twice with PBS, and cultured in serum-free EC medium for another 24 h. The 24-h EC-conditioned medium in the last step was collected individually. After determination of miR-126-3p levels, these EC-conditioned medium were named as "C" (Ctrl siRNA + Ctrl antagomir), "SI" (siERK1/2 + Ctrl antagomir), "A" (Ctrl siRNA + miR-126-3p antagomir) and "A/SI" (siERK1/2 + miR-126-3p antagomir), and used for experiment in Figure S13.

To determine the effect of EC-conditioned medium on calcification in HASMCs, complete DMEM/F12 medium or double-concentrated calcification medium (complete DMEM/F12 medium containing 20 mM β-glycerol phosphate and 500 μM ascorbic acid) was mixed with EC medium, EC-conditioned medium or EC-conditioned medium with aforementioned treatment at 1 : 1 which make the final concentration of β-glycerol phosphate at 10 mM and ascorbic acid at 250 μM. HASMCs in 24-well or 6-well plates were cultured in the above mixed medium or plus U0126 treatment for 7 days to induce calcification. During the course of induction, the culture medium or plus treatment was changed daily. At the end of 7 days treatment, cells in 24-well plate were used to determine cellular calcium deposition by Alizarin Red S staining. Total cellular proteins were extracted from cells cultured in 6-well plates and used to determine expression of BMP2, β-catenin, ALP, LRP6, DKK1 and RUNX2 by Western blot.

Determination of cell viability, cell cycle, proliferation and apoptosis

The 3-(4,5-dimethylthiazol-2-yl)-2,5-diphenyltetrazolium bromide (MTT) colorimetric assay was used to determine cell viability of HUVECs. Briefly, cells in 96-well plate were treated with U0126 or PD98059 at different

concentrations for 18 h. After treatment and aspiration of the treatment medium, cells in each well were added with 40 μL MTT solution (5 mg/mL) and incubated for 4 h. After careful removal of liquid, each well was added with 150 μL DMSO and incubated for 10 min with gentle shake on an orbital shaker to dissolve the purple formazan crystals formed within cells completely. The absorbance of solution at 570 nm in each well was determined by a microplate reader (BioTek, USA). The results were normalized to the mean value of control group and expressed as % of control.

Cell proliferation of VSMCs and ECs in aortic plaques was determined by co-immunofluorescent staining of the corresponding cross sections with anti-SMA or CD31 plus Ki-67 antibody. Cell cycles were determined by FACS assay. Briefly, after treatment, cells were collected and fixed in 70% ethanol for ~24 h. After washed twice with PBS, cells were added with RNase (1 mg/mL) and incubated at 37 °C for 30 min, stained with propidium iodide (PI) solution (2 mg/mL) for 30 min, and then subject to FACS assay.

The early apoptosis of cells was determined by FACS assay. After treatment, cells were staining with annexin-V-PE and 7-aminoactinomycin D (7-AAD) sequentially, then subject to FACS assay. Cells with 7-AAD staining negative while annexin-V-PE staining positive were counted as the cells having early apoptosis.

The late apoptosis of lesions in aortic root was determined by TUNEL assay using the assay kit purchased from Vazyme Biotech Co., Ltd (Nanjing, China) based on instructions from the manufacturer, followed by SMA or CD31 immunofluorescent staining. All images of the cells were obtained with a fluorescence microscope (Leica DM5000B).

Determination of adhesion of THP-1 monocytes to HUVECs

HUVECs in 24-well plates were transfected with miR-126-3p mimic/control mimic or miR-126-3p antagomir/control antagomir for 24 h, followed by culture in complete EC medium for 24 h. Cells were then treated with LPS (100 ng/mL) for 2 h. After removal of the treatment medium, HUVECs were treated with vehicle, U0126 (2 μM) or PD98059 (20 μM) overnight. Meanwhile, THP-1 cells were labeled with carboxyfluorescein succinimidyl ester (CFSE, 5 μM). HUVECs in each well were added same number of CFSE-labeled THP-1 cells and co-cultured for 1 h, followed by washing with PBS for 3 times to remove nonadherent cells. The adherent THP-1 cells were observed and the images of labeled cells were photographed. The number of adherent cells in each sample was counted using the ImageJ software, and normalized by the number in control group (transfected with control mimic/antagomir and no ERK1/2 inhibitor treated), which was defined as 100%.

References

1. Gotoh T, Kuroda T, Yamasawa M, Nishinaga M, Mitsuhashi T, Seino Y, et

al. Correlation between lipoprotein(a) and aortic valve sclerosis assessed by echocardiography (the JMS Cardiac Echo and Cohort Study). Am J Cardiol. 1995; 76: 928-32.
2. Agatston AS, Janowitz WR, Hildner FJ, Zusmer NR, Viamonte M, Jr., Detrano R. Quantification of coronary artery calcium using ultrafast computed tomography. J Am Coll Cardiol. 1990; 15: 827-32.
3. Chen Y, Duan Y, Yang X, Sun L, Liu M, Wang Q, et al. Inhibition of ERK1/2 and activation of LXR synergistically reduce atherosclerotic lesions in ApoE-deficient mice. Arterioscler Thromb Vasc Biol. 2015; 35: 948-59.
4. Zernecke A, Bidzhekov K, Noels H, Shagdarsuren E, Gan L, Denecke B, et al. Delivery of microRNA-126 by apoptotic bodies induces CXCL12-dependent vascular protection. Sci Signal. 2009; 2: ra81.
5. Schmittgen TD, Jiang J, Liu Q, Yang L. A high-throughput method to monitor the expression of microRNA precursors. Nucleic Acids Res. 2004; 32: e43.
6. Liu L, Zeng P, Yang X, Duan Y, Zhang W, Ma C, et al. Inhibition of Vascular Calcification: a new antiatherogenic mechanism of Topo II (DNA Topoisomerase II) inhibitors. Arterioscler Thromb Vasc Biol. 2018; 38: 2382-2395.
7. Chen Y, Duan Y, Kang Y, Yang X, Jiang M, Zhang L, et al. Activation of liver X receptor induces macrophage interleukin-5 expression. J Biol Chem. 2012; 287: 43340-50.
8. Zhang B, Zhang Z, Xia S, Xing C, Ci X, Li X, et al. KLF5 activates microRNA 200 transcription to maintain epithelial characteristics and prevent induced epithelial-mesenchymal transition in epithelial cells. Mol Cell Biol. 2013; 33: 4919-35.

II. Supplementary table

Table S1. Sequences of primers for qRT-PCR

Gene	Forward	Backward
AMPKα1	ACCAGGTCATCAGTACACCA	GGACCACCATATGCCTGTGA
AMPKα2	GGCAAAGTGAAGATTGGAGAACA	CCGTCCATGCTTACAGATGTAGT
DKK1	TTCCATTTTTGCAGTAATTCCC	AGTACTGCGCTAGTCCCACC
Drosha	GTTTCCAAACCGCAACCGAG	CCGGGAAAAGCAACCTGGAA
EGFL7	GGATGACTGATTCTCCTCCG	CAACACCAGAAGCCACATCA
GAPDH	GGTGGTCTCCTCTGACTTCAACA	GTTGCTGTAGCCAAATTCGTTGT
LRP5	GAGATCCTCCGTAGGTCCGT	CCAAGCGAGCCTTTCTACAC
LRP6	TCAGTCCATTTGGCCAGTAA	CAACCCAGAGCTATTGCCTT
Osx	GGGACTGGAGCCATAGTGAA	CTCAGCTCTCTCCATCTGCC
pri-miR-126	TATCAGCCAAGAAGGCAGAA	CGTGGCGTCTTCCAGAAT
pre-miR-126	GCTGGCGACGGGACATTAT	TGCCGTGGACGGCGCATTA
p53	GCTCGACGCTAGGATCTGAC	GCTTTCCACGACGGTGAC
RUNX2	CCTAAATCACTGAGGCGGTC	CAGTAGATGGACCTCGGGAA
SOX9	GTGGTCCTTCTTGTGCTGC	GTACCCGCACTTGCACAAC
USF1	ATCCGAGGAACTGGTCCTTT	GAGATACCTAGGCCGGGAGA
U6	CTCGCTTCGGCAGCACA	AACGCTTCACGAATTTGCGT

DKK1: dickkopf 1; EGFL7: EGF like domain multiple 7; GAPDH: glyceraldehyde-3-phosphate dehydrogenase; LRP5/6: LDL receptor related protein 5 or 6; Osx: osterix; pri-miR-126: primary miR-126; pre-miR-126: miR-126 precursor; RUNX2: runt related transcription factor 2; SOX9: sex-determining region y-box 9; USF1: upstream transcription factor 1.

Table S2. Basal clinical characteristics of CAVD patients

Clinical characteristics	CAVD patients (n = 15)
Age (years)	68.5 ± 4.0
Sex (Male/female)	8/7
BMI (kg/m^2)	25.4 ± 2.0
Smoking	6 (40)
Cerebrovascular disease	1 (6.7)
Coronary heart disease	1 (6.7)
Hypertension	8 (53.3)
Diabetes mellitus	4 (26.7)
Dyslipidemia	9 (60)
AVA (cm^2)	0.8 ± 0.1
Aorta Vmax (m/s)	5.5 ± 0.6
P-mean (mm Hg)	70.1 ± 11.5
AVC score	324.5 - 494.5

Values are shown as mean ± SD, n (%) or interquartile range. CAVD, calcified aortic valve disease; BMI, body mass index; AVA, the aortic valve area;

Aorta Vmax, the maximum aortic velocity; P-mean, the mean transvalvular pressure gradient; AVC, aortic valve calcification.

III. Supplementary figures

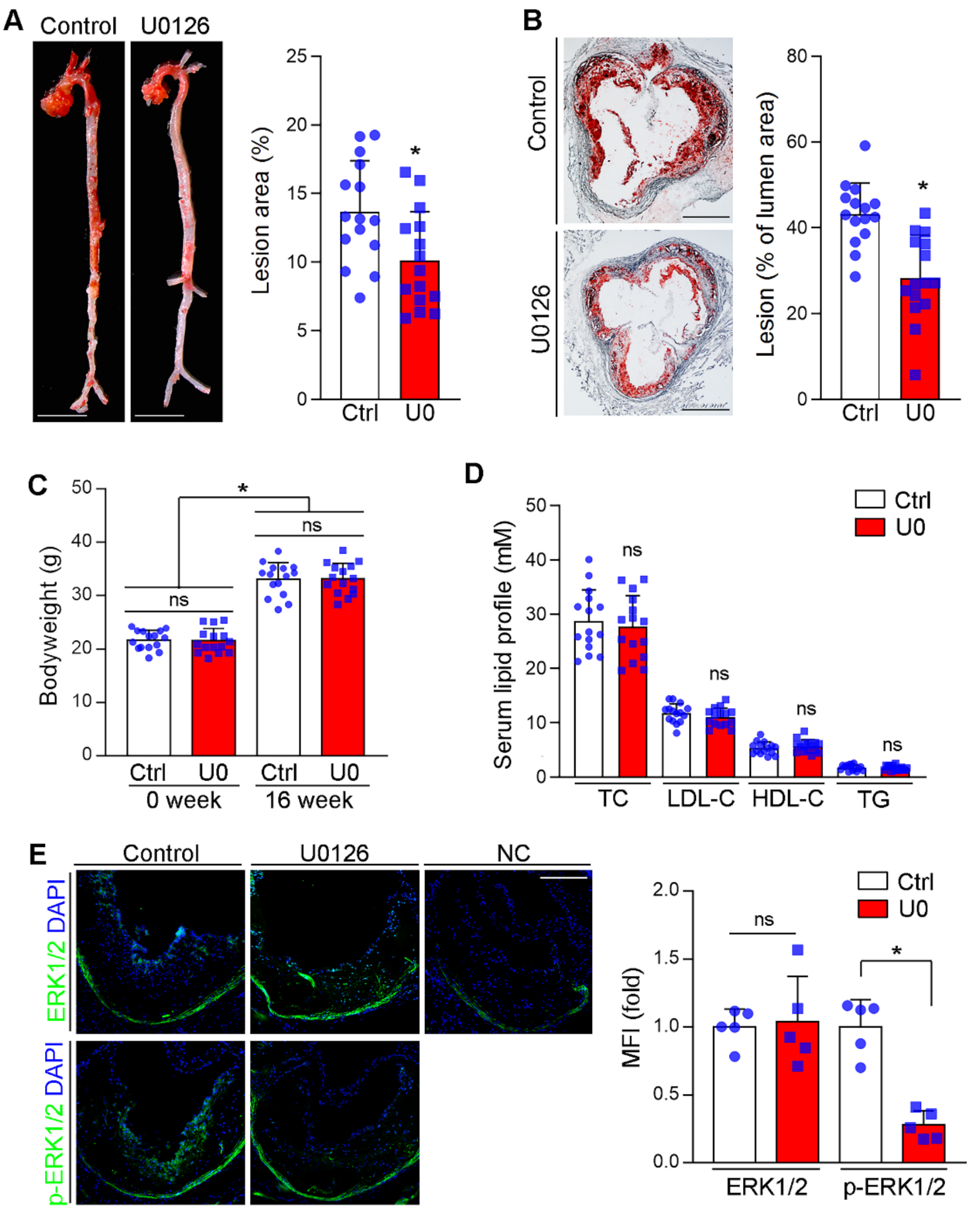

Figure S1. U0126 inhibits atherosclerosis in apoE$^{-/-}$ mice by inhibiting p-ERK1/2

ApoE$^{-/-}$ mice (15 mice/group) were fed high-fat diet (HFD) or HFD containing U0126 (3 mg/kg bodyweight/day) for 16 weeks. Aortas were collected for determination of lesions in *en face* aortas (A, Bars: 5 mm) and sinus of aortic root (B, Bars: 400 μm) by Oil red O staining with quantitative analysis of lesion areas. (C) mouse bodyweight was determined at the beginning and the end of experiment. (D) serum TC, LDL-C, HDL-C, and TG content were measured at the end of experiment. *P < 0.05; ns: not significant (n = 15). (E) expression of ERK1/2 and p-ERK1/2 in aortas were determined by immunofluorescent staining of the sections with quantitation of MFI. *P < 0.05, ns: not significantly

difference (n = 5). Bars: 200 μm.

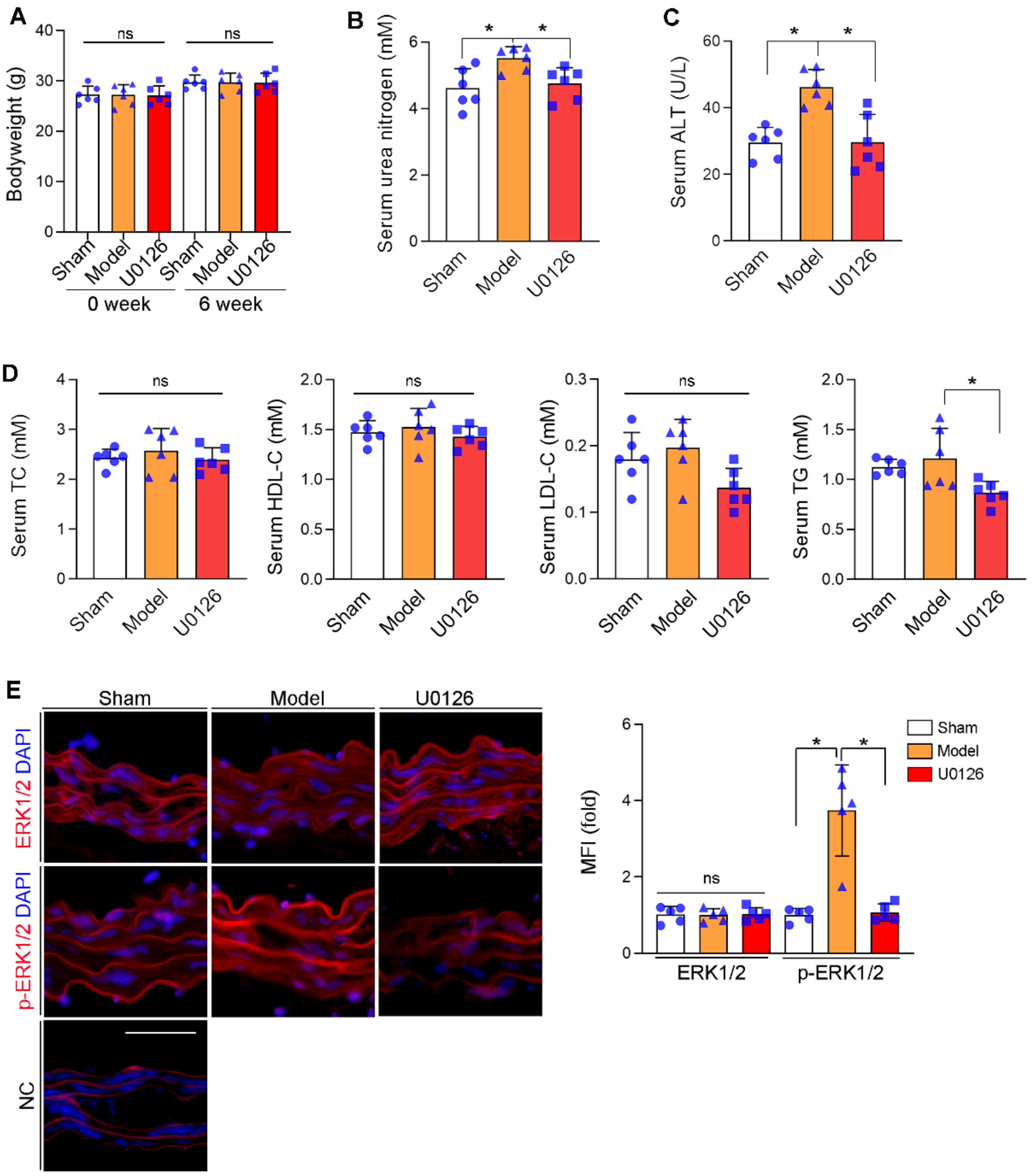

Figure S2. U0126 attenuates VD_3-induced metabolic disorders in kidney and liver and p-ERK1/2 in aortas

C57BL/6J mice (6 mice/group) were s.c. injected with 100 μL olive oil (Sham) or VD_3 dissolved in olive oil ($5.5x10^5$ U/kg) every day for three days, followed by feeding with chow diet (Model) or chow diet containing U0126 (3 mg/kg bodyweight/day) (U0126) for 6 weeks. Mouse bodyweight was determined at the beginning and the end of experiment (A); serum urea nitrogen (B), ALT (C), TC, HDL-C, LDL-C and TG content (D) were determined, respectively. *P < 0.05 (n = 6). (E) expression of ERK1/2 and p-ERK1/2 in thoracic aortas were determined by immunofluorescent staining of the sections with quantitation of MFI, Bars: 200 μm. *P < 0.05, ns: not significantly difference (n = 5).

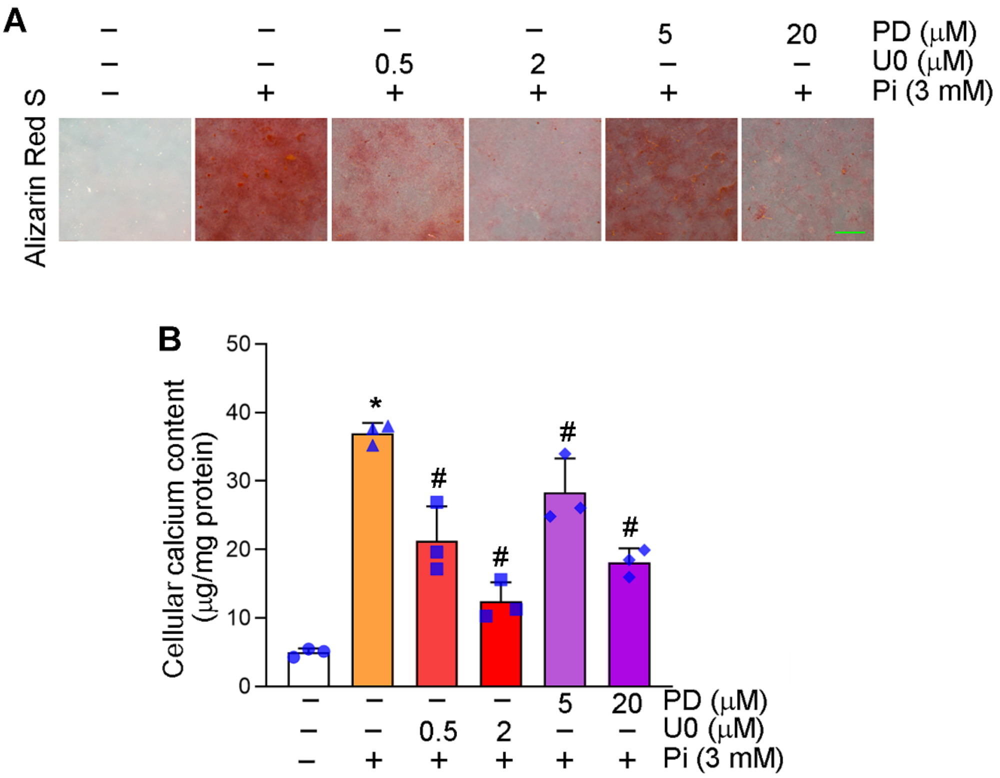

Figure S3. ERK1/2 inhibitors reduce inorganic phosphate-induced calcification in HASMCs

HASMCs cultured in complete DMEM/F12 medium or complete DMEM/F12 medium containing 3 mM inorganic phosphate (Na_2HPO_4/NaH_2PO_4) or plus PD98059 or U0126 at the indicated concentrations for 7 days. Cellular calcium content was determined by Alizarin Red S staining (A) and calcium quantitative analysis, which was normalized by cellular protein content (B). *P < 0.05 *vs*. complete DMEM/F12 medium; $^{\#}$P < 0.05 *vs*. the medium containing inorganic phosphate (n = 3). Green bar: 0.5 mm.

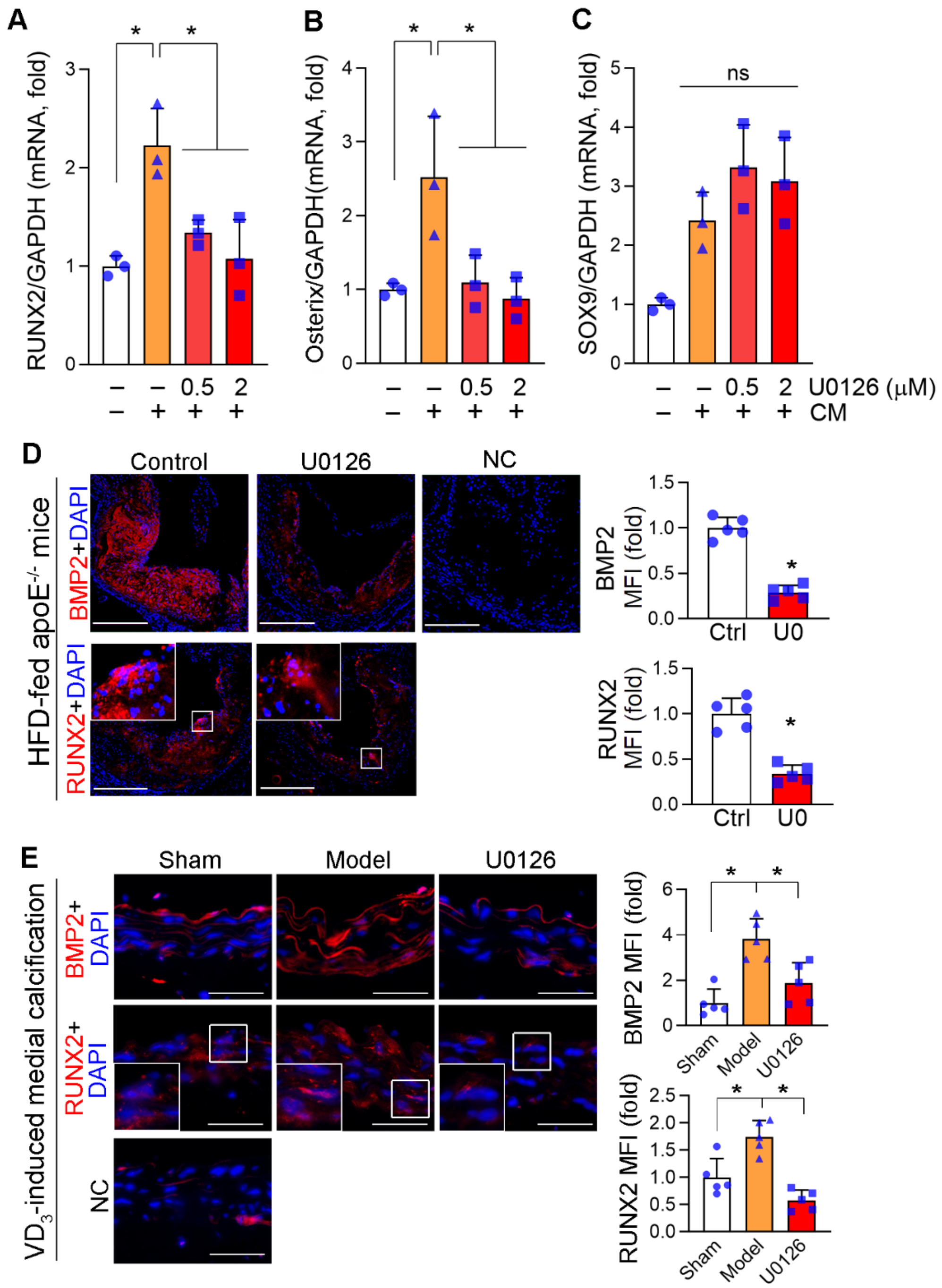

Figure S4. U0126 inhibits RUNX2, Osx and BMP2 expression *in vitro* and *in vivo*

(A-C) HASMCs were cultured in complete DMEM/F12 medium, calcification medium (CM) or CM plus U0126 treatment at the indicated concentrations for 7 days. Expression of RUNX2 (A), Osterix (B) and SOX9 (C) mRNA were determined by qRT-PCR. *P < 0.05, ns: not significant (n = 3). (D-E) the aortic root and thoracic aortic cross sections were prepared from mice used in Figure S1 and Figure 2D. Expression of BMP2 and RUNX2 in aortas were determined by immunofluorescent staining of the sections with quantitation of MFI. *P < 0.05 (n = 5). Bars: 200 μm (D), 50 μm (E).

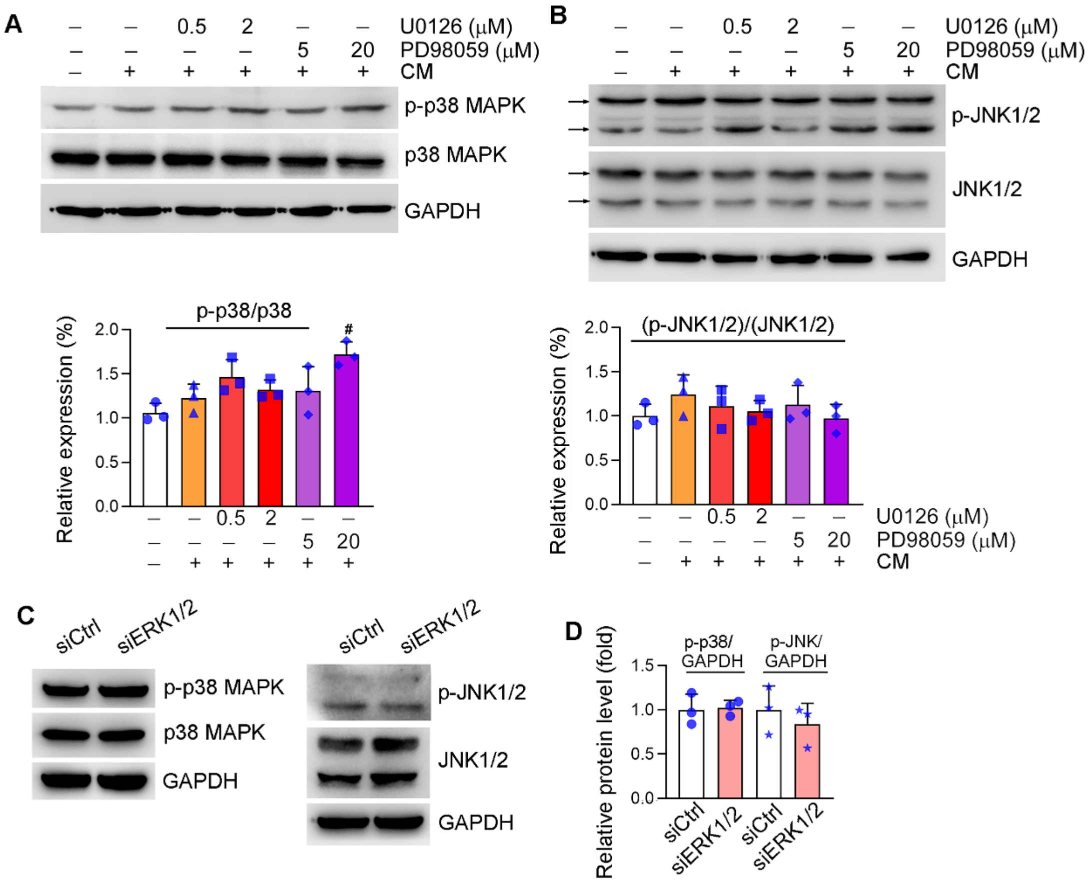

Figure S5. ERK1/2 inhibition does not affect activity of p38 MAPK and JNK1/2

(A-B) HASMCs were cultured in complete DMEM/F12 (1 : 1) medium or calcification medium (CM) or CM containing U0126 or PD98059 at the indicated concentrations for 7 days. Activated p38 MAPK (p-p38 MAPK), p38 MAPK, activated JNK1/2 (p-JNK1/2) and JNK1/2 protein levels were determined by Western blot (A). The ratios of band density of p-p38 MAPK to p38 MAPK and p-JNK1/2 to JNK1/2 were quantitatively analyzed (B). #P < 0.05 *vs.* CM control (n = 3). (C-D) HASMCs were transfected with control or ERK1/2 siRNA for 24 h. Cells were then switched to complete DMEM/F12 (1 : 1) medium and cultured for another 48 h. After treatment, total cellular proteins were extracted and used to determine expression of p-p38 MAPK, p38 MAPK, p-JNK1/2 and JNK1/2 by Western blot with quantitative analysis of band density.

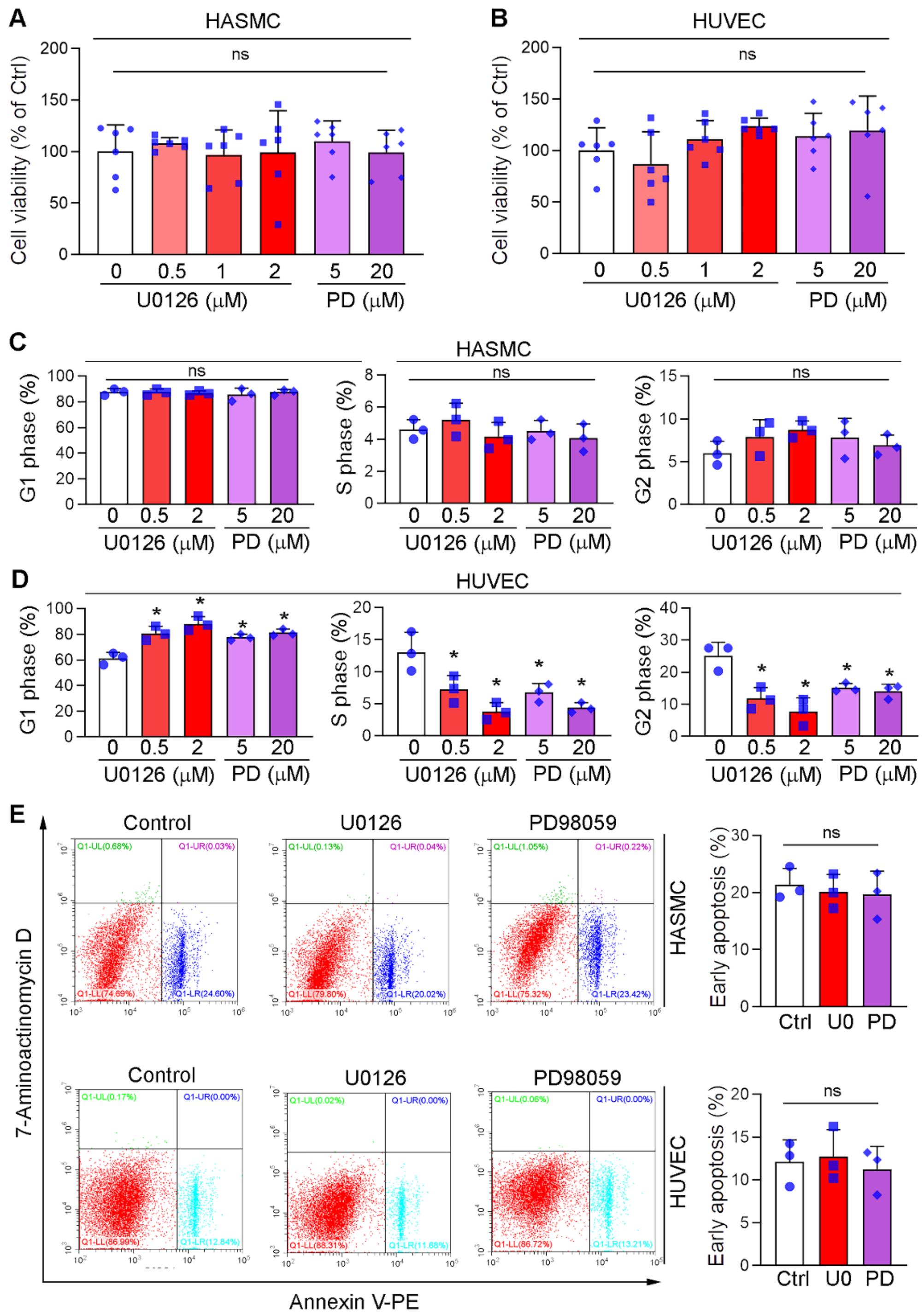

Figure S6. ERK1/2 inhibition has no effect on viability and apoptosis of HASMCs and HUVECs, but moderately reduces G1/S transition in HUVECs

(A-D) HASMCs (4x10^4 cells/well) or HUVECs (5x10^4 cells/well) in 96-well plates were treated with U0126 or PD98059 at the indicated concentrations for 18 h. Cell viability was determined using MTT method (A, B, n = 6); Cell cycle was determined using FACS assay (C-D, n = 3). (E) both HASMCs and HUVECs were treated with U0126 (2 μM) and PD98059 (20 μM) for 18 h, followed by determination of early apoptosis using FACS assay (n = 3).

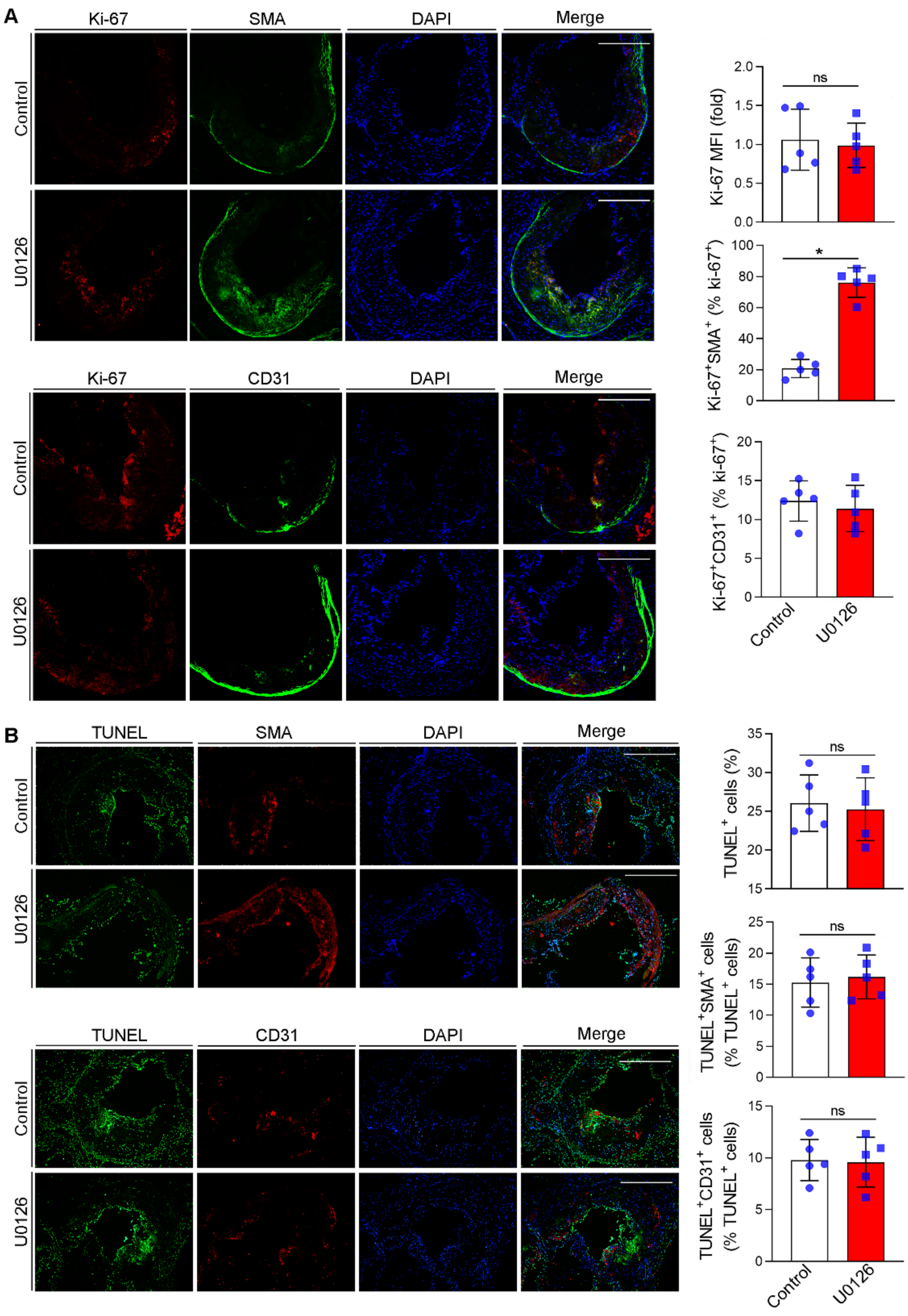

Figure S7. U0126 increases SMC proliferation while does not affect EC proliferation or apoptosis of SMCs and ECs in mouse aortic plaque

(A) cross sections of mouse aortic root used in Figure S1 were conducted co-immunofluorescent staining with anti-SMA or CD31 plus Ki-67 antibodies with quantitation of Ki-67, Ki-67^{+}SMA^{+}, Ki-67^{+}CD31^{+} MFI (n = 5). (B) the sections were also used to conduct immunofluorescent staining with anti-SMA or CD31 antibody followed by TUNEL assay. Total TUNEL positive cells, TUNEL^{+}SMA^{+} cells, TUNEL^{+}CD31^{+} cells were quantitatively analyzed (B, n = 5). Bars: 200 μm.

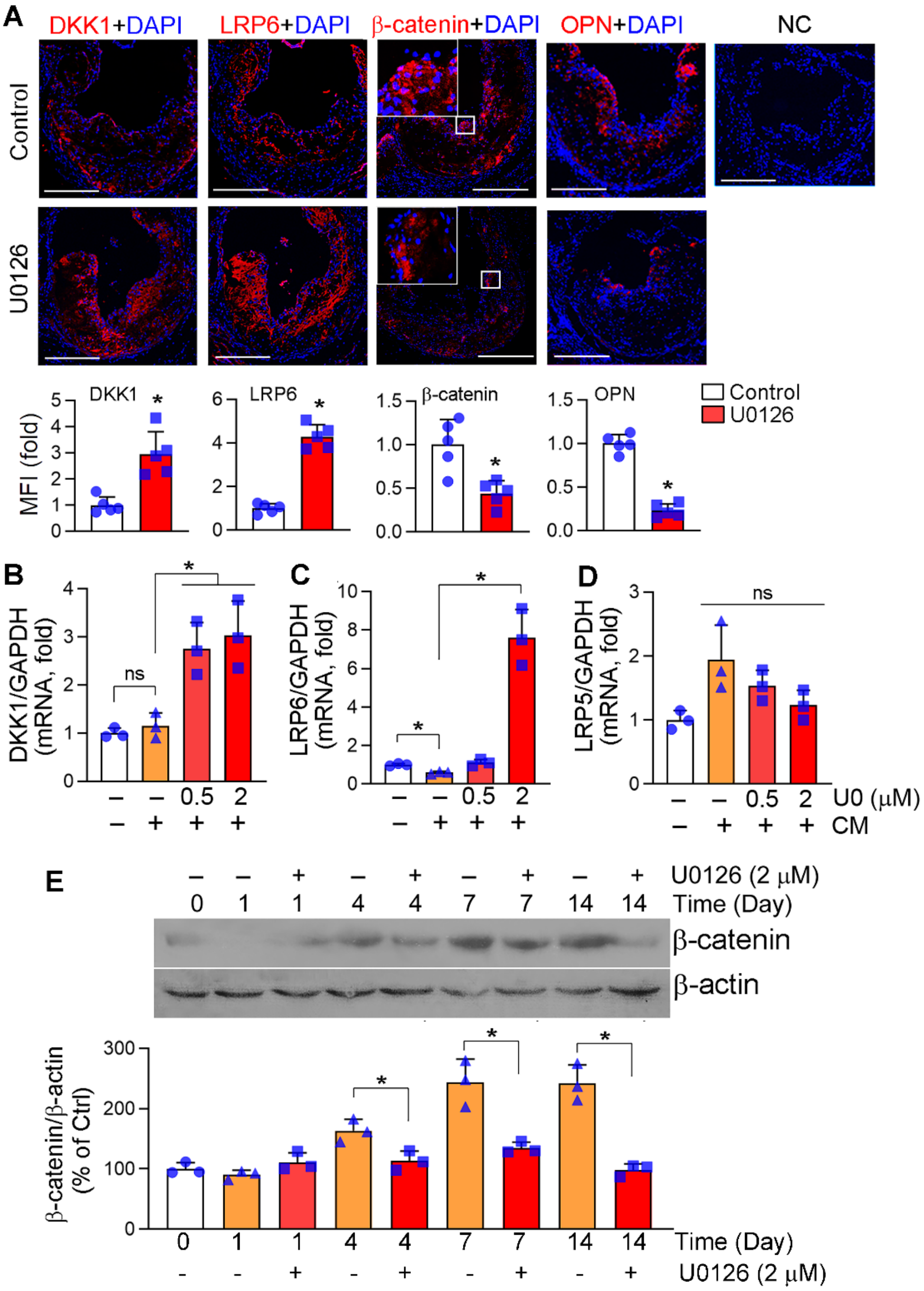

Figure S8. ERK1/2 inhibition inactivates Wnt signaling pathway

(A) the aortic root cross sections prepared from the mice used in Figure S1 were used to determine expression of DKK1, LRP6, β-catenin and OPN in atherosclerotic plaque area by immunofluorescent staining with quantitation of MFI. Bars: 200 μm. *P < 0.05 (n = 5). (B-D) HASMCs were cultured in complete DMEM/F12 medium, calcification medium (CM) or CM plus U0126 treatment at the indicated concentrations for 7 days. Expression of DKK1 (B), LRP6 (C) and LRP5 (D) mRNA were determined by qRT-PCR. *P < 0.05; ns: not significant (n = 3). (E) HASMCs cultured in CM were treated with 2 μM U0126 for the indicated times. Expression of β-catenin protein was determined

by Western blot with total cellular proteins. *P < 0.05 *vs*. corresponding control (without U0126 treatment at the same time point), n = 3.

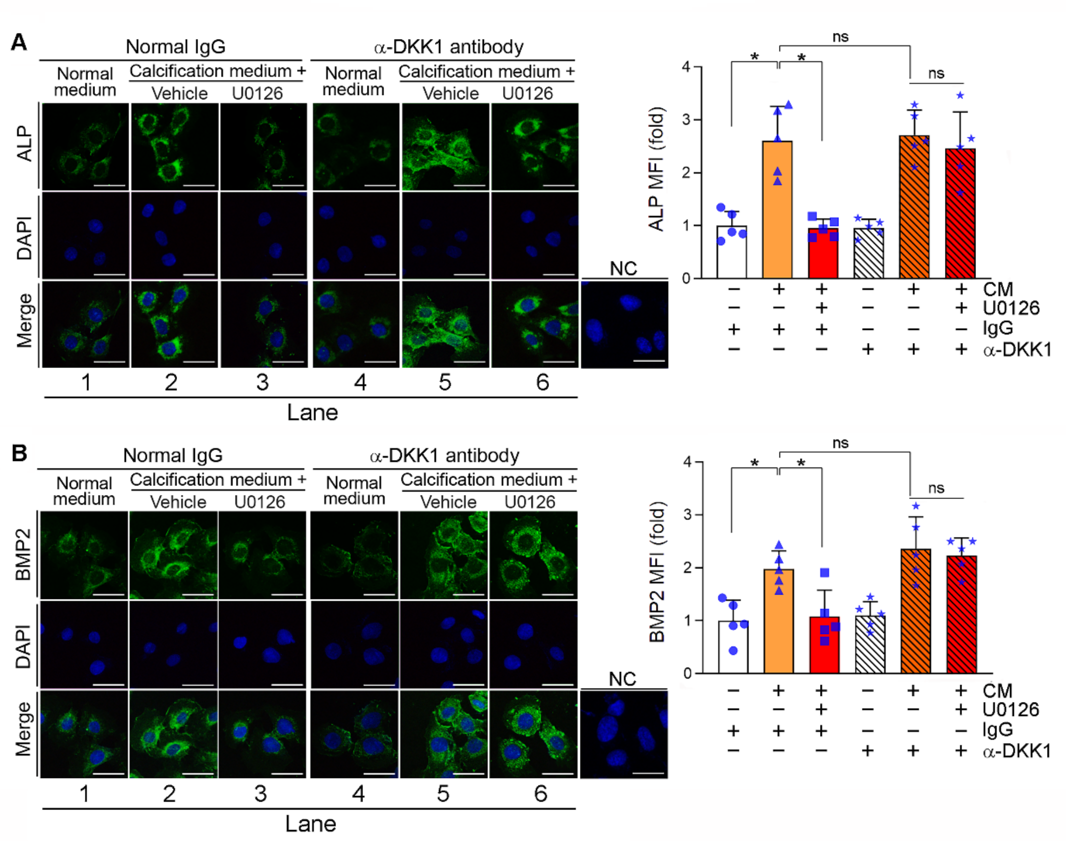

Figure S9. DKK1 antibody attenuates inhibitory effects of U0126 on calcification-induced BMP2 and ALP expression in HASMCs

HASMCs cultured in complete DMEM/F12 medium or calcification medium (CM) were treated with U0126 (2 μM) in the presence of normal IgG or anti-DKK1 antibody for 7 days. Expression of BMP2 (A) and ALP (B) were determined by immunofluorescent staining with quantitation of MFI. *P < 0.05, ns: not significant (n = 3). Bars: 20 μm.

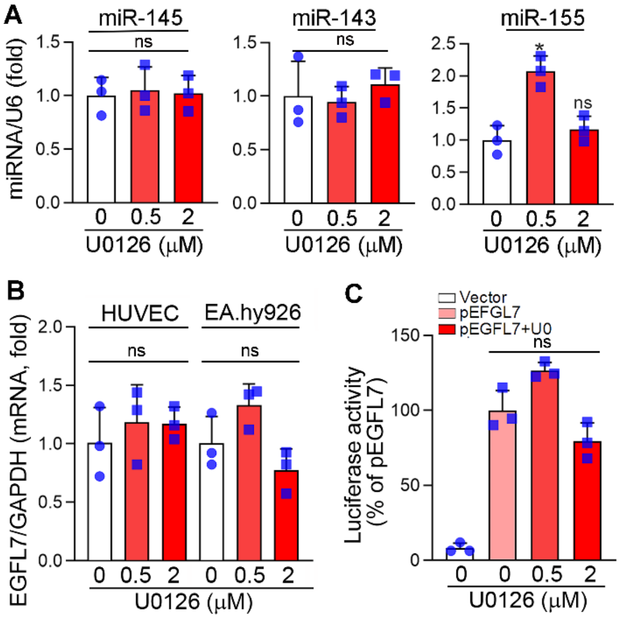

Figure S10. ERK1/2 inhibition regulates EC miRNA expression while has no effect on EGFL7 expression

(A) HUVECs were treated with U0126 at the indicated concentrations for 16 h. Expression of miR-145, miR-143 and miR-155 were determined by miR stem-loop RT-PCR technology (n = 3). (B) HUVECs or EA.hy926 cells were treated with U0126 at the indicated concentrations for 16 h. Expression of EGFL7 mRNA was determined by qRT-PCR (n = 3). (C) EA.hy926 cells in 24-well plates were co-transfected with empty vector or EGFL7 promoter (pEGFL7) containing *Firefly* luciferase plus vector containing *Renilla* luciferase as reference for 4 h, followed by U0126 treatment at the indicated concentrations overnight and determination of activities of *Firefly* and *Renilla* luciferases (n = 3).

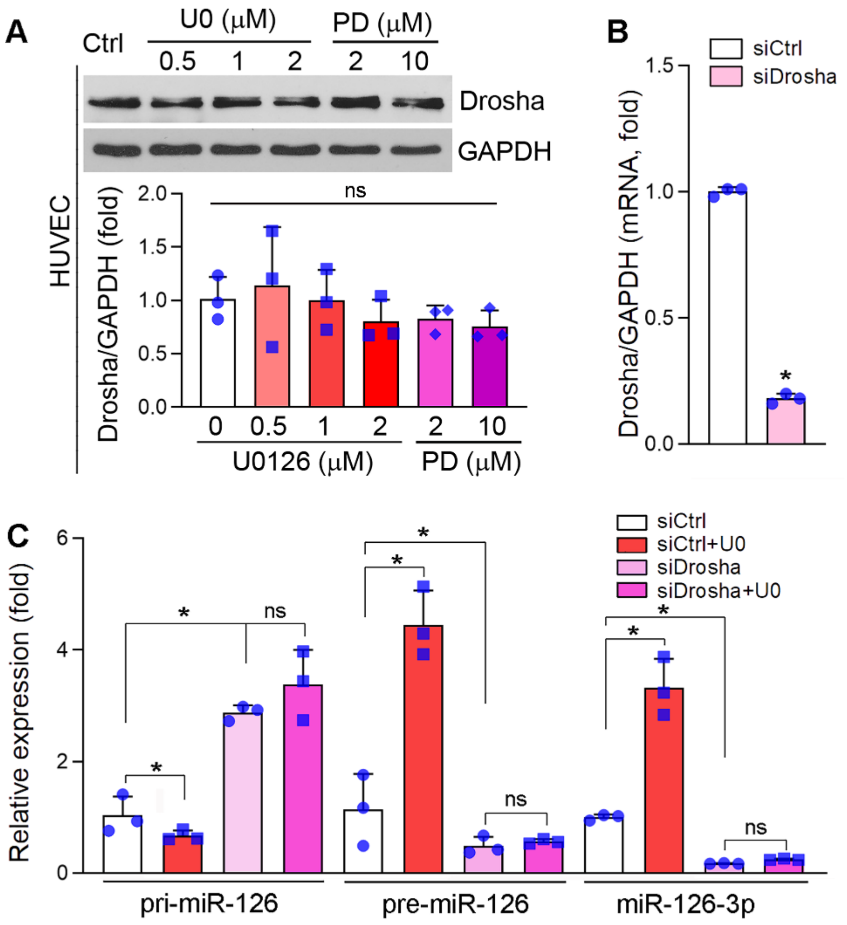

Figure S11. ERK1/2 inhibition-enhanced miR-126-3p maturation is independent of Drosha protein expression

(A) HUVECs were treated with U0126 and PD98059 at the indicated concentrations overnight. Drosha protein level was determined by Western blot (n = 3). (B) HUVECs were transfected with control or Drosha siRNA for 24 h, followed by culture in complete medium for another 24 h. Expression of Drosha mRNA level was determined by qRT-PCR. *$P < 0.05$; (n = 3). (C) HUVECs were transfected with control or Drosha siRNA for 24 h, followed by culture in complete medium for another 24 h. Cells were then treated with U0126 at 2 μM for 12 h, followed by determination of pri-miR-126, pre-miR-126 by qRT-PCR and miR-126-3p levels by miR stem-loop RT-PCR technology. *$P < 0.05$ (n = 3).

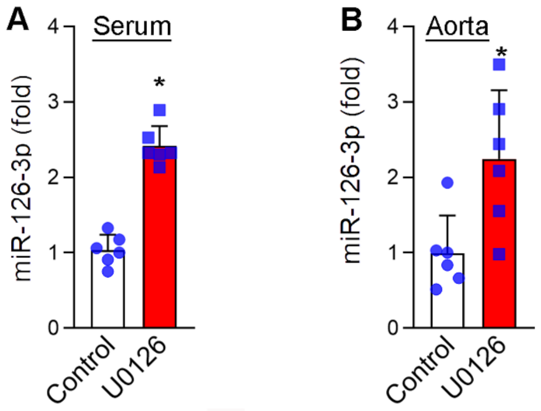

Figure S12. U0126 increases serum and aortic miR-126-3p expression
Serum and aortic samples collected from mice used in Figure S1 were used to determine miR-126-3p levels in serum (A) and aorta (B) by miR stem-loop RT-PCR technology. *P < 0.05 (n = 6).

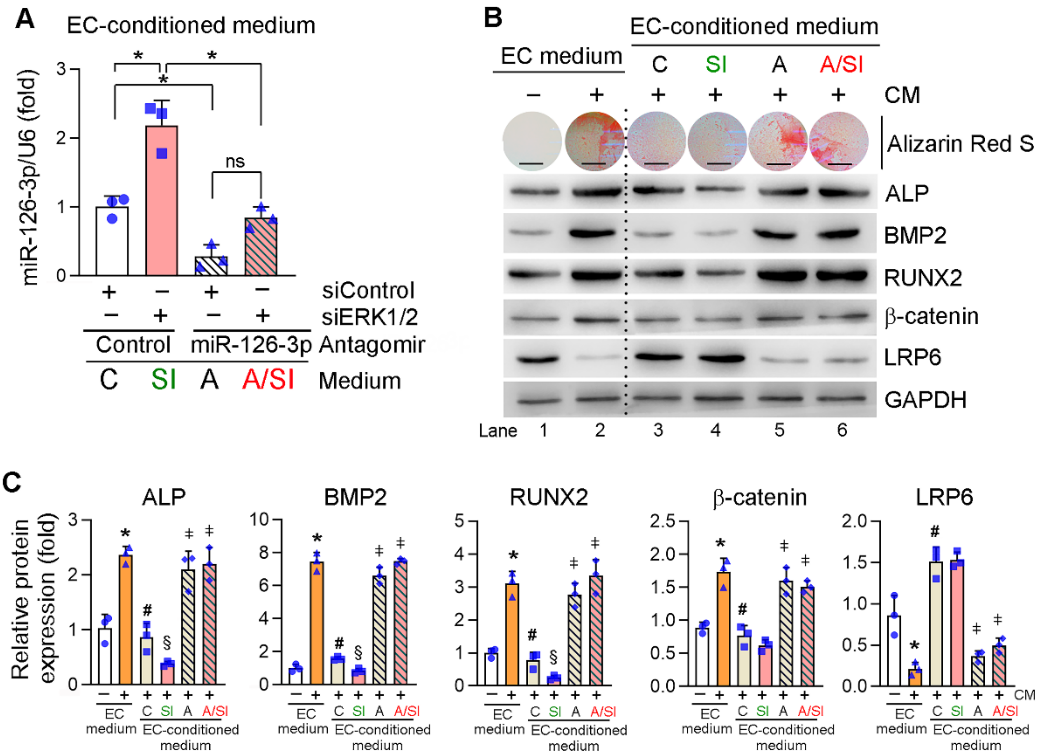

Figure S13. ERK1/2 siRNA activates EC miR-126-3p production/secretion to inhibit HASMC calcification

(A) HUVECs were co-transfected with control antagomir or miR-126-3p antagomir plus control siRNA or ERK1/2 siRNA as indicated. After 24 h of co-transfection, cells were switched to complete medium for 24 h, followed by culture in serum-free EC medium for another 24 h. The later 24-h EC-conditioned medium was collected individually and determined miR-126-3p levels by miR stem-loop RT-PCR technology. *P < 0.05; ns: not significant (n = 3). The conditioned medium collected from control siRNA plus control antagomir-, siERK1/2 plus control antagomir-, control siRNA plus antago-miR-126-3p-, or siERK1/2 plus antago-miR-126-3p-transfected ECs was named as "C", "SI", "A" or "A/SI" medium separately. (B-C) HASMCs were cultured in complete DMEM/F12 medium, concentrated CM mixed with EC medium (1 : 1) (sample #1, 2); or concentrated CM mixed with the collected "C", "SI", "A", or "A/SI" EC-conditioned medium above (1 : 1, sample #3-6). All the medium (sample #1-6) were changed daily. After 7 days of treatment, cellular calcium deposit was determined by Alizarin Red S staining. Bars: 5 mm. Expression of ALP, BMP2, RUNX2, β-catenin and LRP6 in total cellular proteins were determined by Western blot (B) with statistical results of band density analysis (C). *P < 0.05 *vs.* lane 1, $^{\#}$P < 0.05 *vs.* lane 2, §P < 0.05 *vs.* lane 3, ‡P < 0.05 *vs.* lane 4 (n = 3).

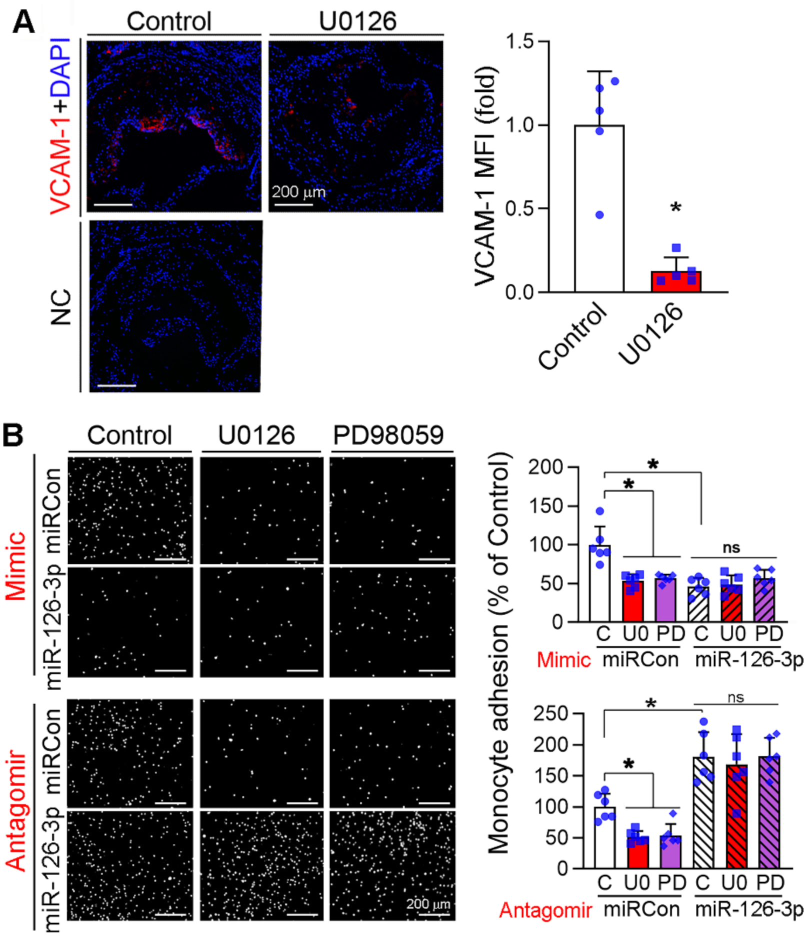

Figure S14. Activated EC miR-126-3p by ERK1/2 inhibitors reduces adhesion of monocytes to endothelial cells

(A) the aortic root cross sections prepared from the mice used in Figure S1 were used to determine VCAM-1 expression in atherosclerotic plaque area by immunofluorescent staining with quantitation of MFI. *P < 0.05 (n = 5). (B) HUVECs were transfected with control or miR-126-3p mimic/antagomir as indicated for 24 h, followed by culture in complete EC medium for 24 h. Cells were then treated with LPS (100 ng/mL) for 2 h. After removal of LPS, HUVECs were treated with vehicle (Control or C), U0126 (U0, 2 µM) or PD98059 (PD, 20 µM) overnight. HUVECs were then added with CFSE-labeled THP-1 monocytes and co-cultured for 1 h. The non-adherent cells were removed by washing with PBS. The images of adherent THP-1 cells to HUVECs were captured with a microscope and counted. The number of total adherent cells in sample transfected with control mimic or antagomir plus LPS treatment was defined as 100%. *P < 0.05; ns: not significant (n = 6).

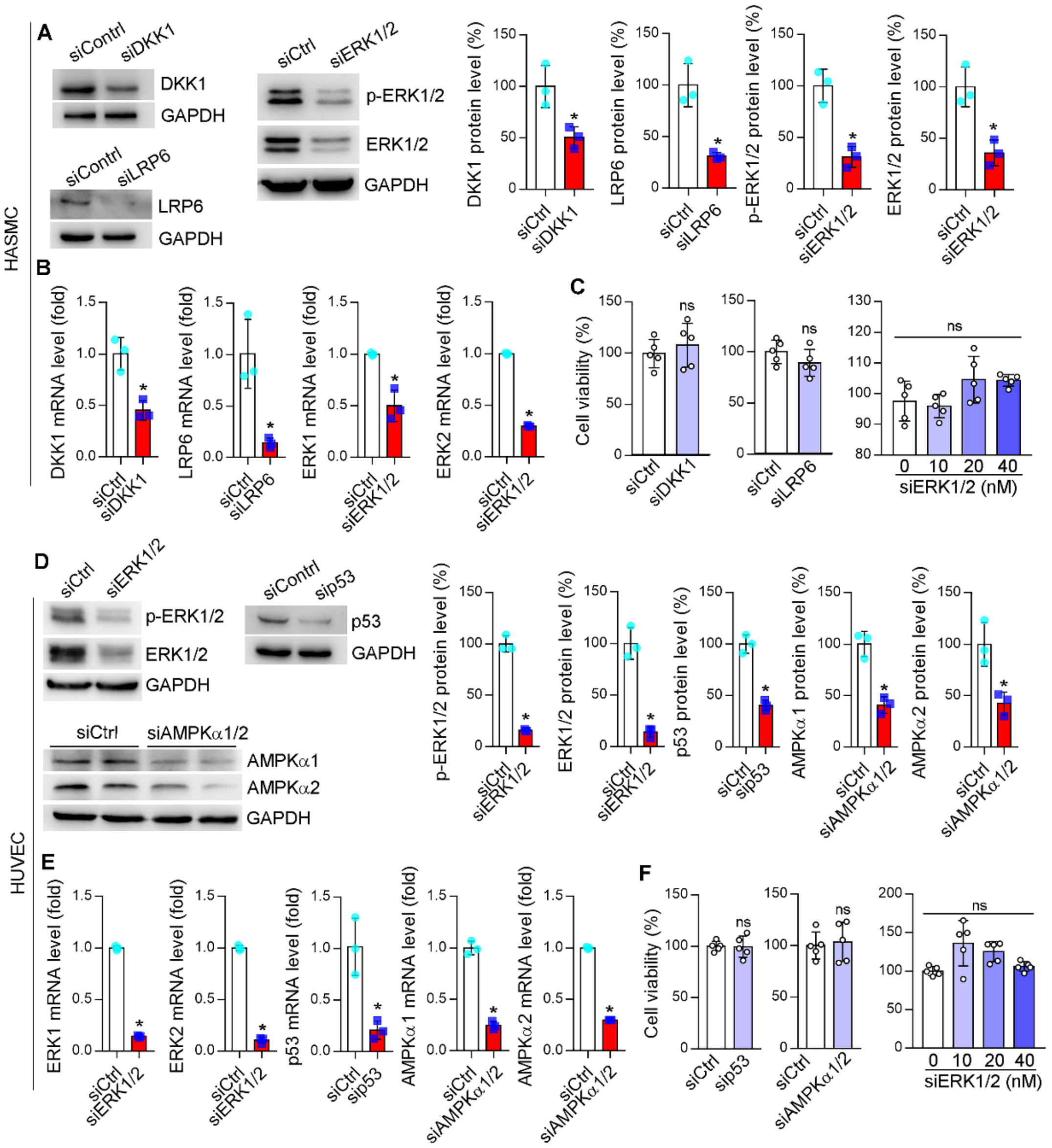

Figure S15. Inhibtion of target gene expression by siRNA has no effect on cell viability

HASMCs or HUVECs were transfected with the indicated siRNA or control siRNA for 24 h. Cells were then cultured in complete medium for 48 h, followed by determination of target gene protein/mRNA expression by Western blot/qRT-PCR (A-B, D-E). *P < 0.05 (n = 3), or cell viability by MTT assay (C, F). ns: not significant (n = 5).

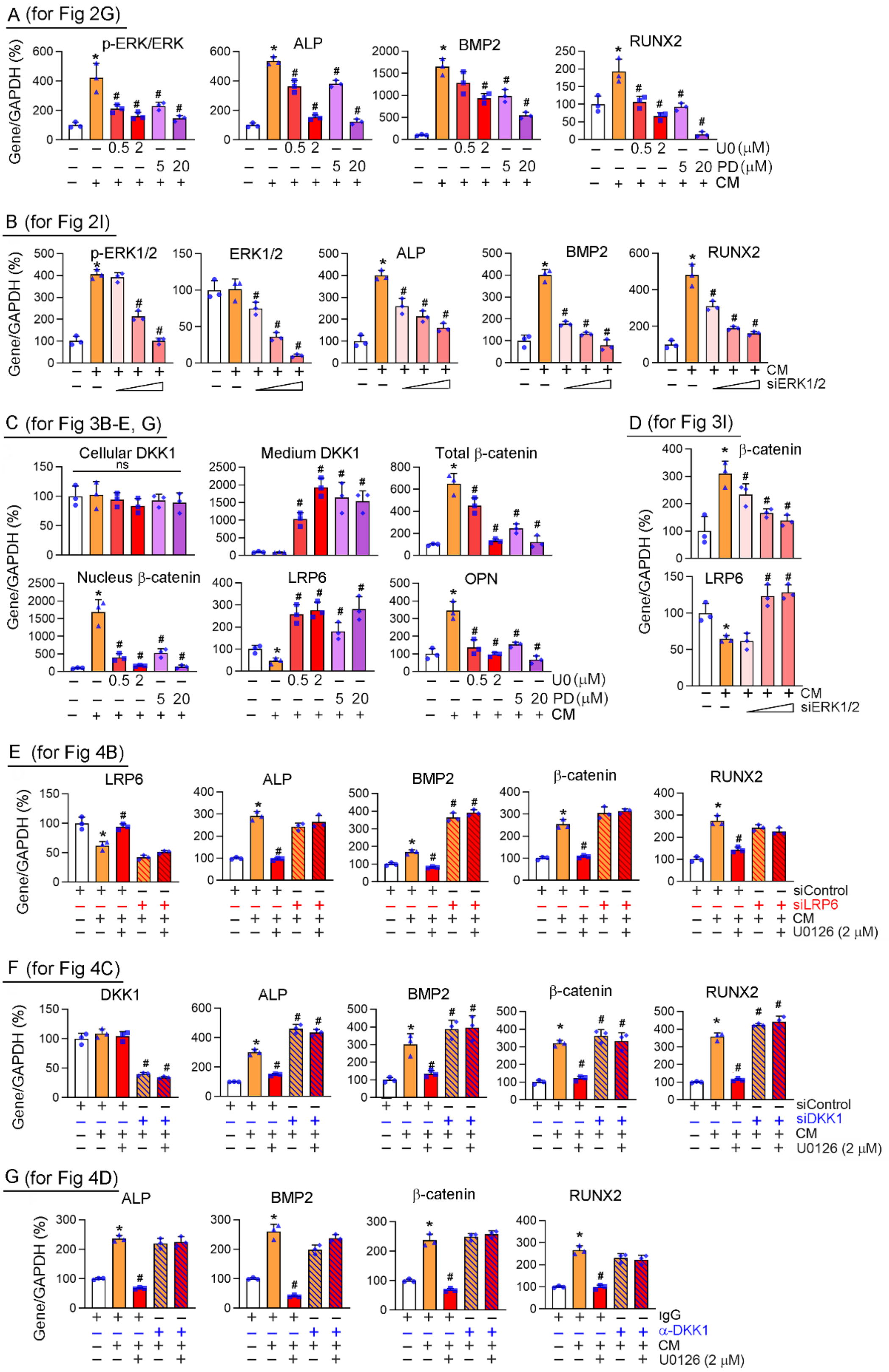
A (for Fig 2G)
p-ERK/ERK
ALP
BMP2
RUNX2
Gene/GAPDH (%)
U0 (μM)
PD (μM)
CM
B (for Fig 2I)
p-ERK1/2
ERK1/2
ALP
BMP2
RUNX2
CM
siERK1/2
C (for Fig 3B-E, G)
Cellular DKK1
ns
Medium DKK1
Total β-catenin
Nucleus β-catenin
LRP6
OPN
U0 (μM)
PD (μM)
CM
D (for Fig 3I)
β-catenin
LRP6
CM
siERK1/2
E (for Fig 4B)
LRP6
ALP
BMP2
β-catenin
RUNX2
siControl
siLRP6
CM
U0126 (2 μM)
F (for Fig 4C)
DKK1
ALP
BMP2
β-catenin
RUNX2
siControl
siDKK1
CM
U0126 (2 μM)
G (for Fig 4D)
ALP
BMP2
β-catenin
RUNX2
IgG
α-DKK1
CM
U0126 (2 μM)

(continued)

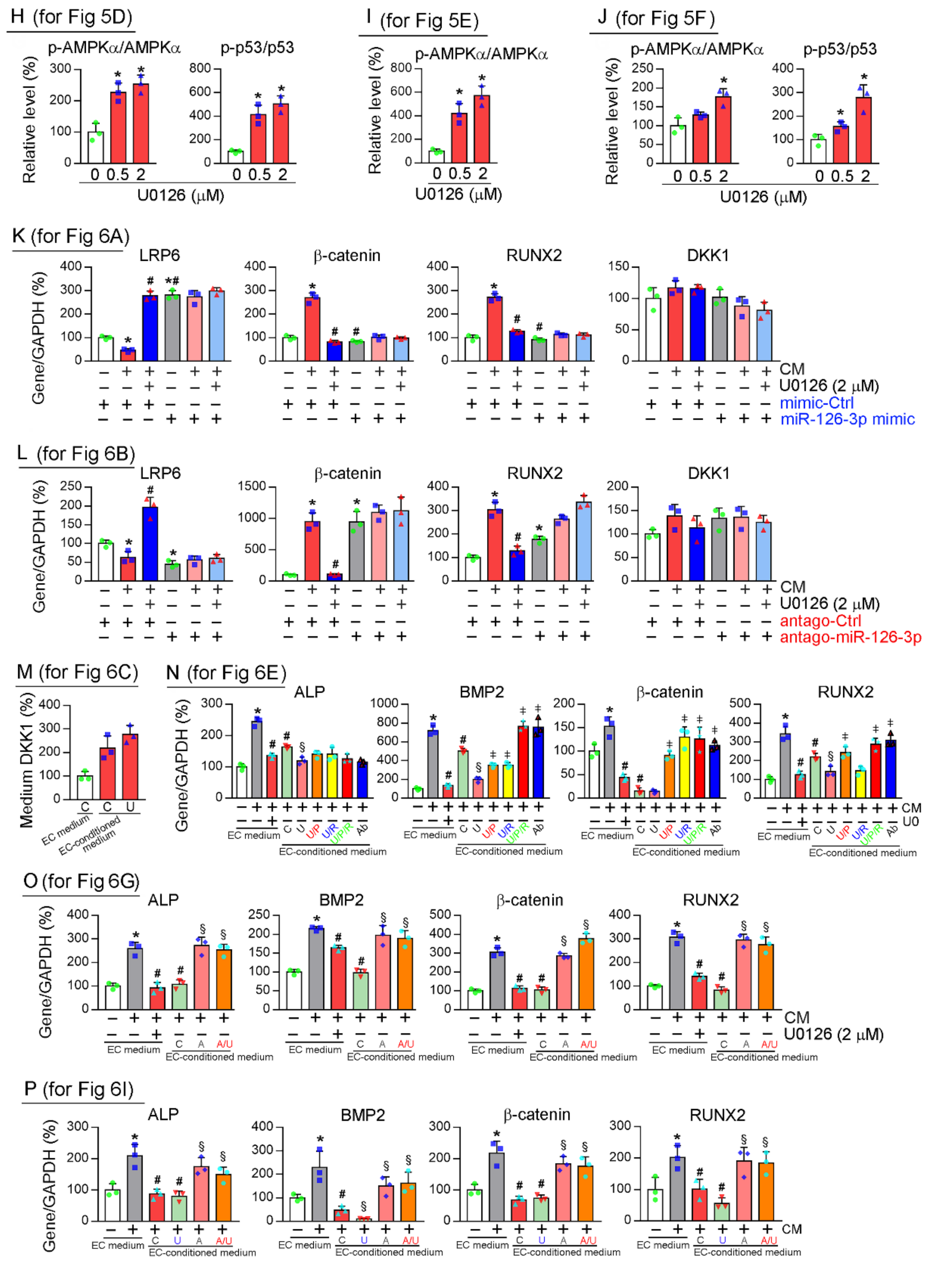

Figure S16. The results of statistical analysis of band density for Western blots in Figure 2-6

All the images of Western blots were conducted band density analysis. The statistical results of mean band density were listed in the below of the corresponding figure. The statistical results were also used to make the dot-like graphs as shown in this figure. (A) Figure 2G. (B) Figure 2I; (C) Figure 3B-3E, 3G. (D) Figure 3I. (E) Figure 4B. (F) Figure 4C. (G) Figure 4D. (H)

Figure 5D. (I) Figure 5E. (J) Figure 5F. (K) Figure 6A. (L) Figure 6B. (M) Figure 6C. (N) Figure 6E. (O) Figure 6G. (P) Figure 6I. *, #, §, ‡P < 0.05 as indicated in the individual figure legends (n = 3).
